# Enabling genome editing in tropical maize lines through an improved, morphogenic regulator-assisted transformation protocol

**DOI:** 10.3389/fgeed.2023.1241035

**Published:** 2023-12-07

**Authors:** José Hernandes-Lopes, Maísa Siqueira Pinto, Letícia Rios Vieira, Patrícia Brant Monteiro, Sophia V. Gerasimova, Juliana Vieira Almeida Nonato, Maria Helena Faustinoni Bruno, Alexander Vikhorev, Fernanda Rausch-Fernandes, Isabel R. Gerhardt, Laurens Pauwels, Paulo Arruda, Ricardo A. Dante, Juliana Erika de Carvalho Teixeira Yassitepe

**Affiliations:** ^1^ Genomics for Climate Change Research Center (GCCRC), Universidade Estadual de Campinas, Campinas, Brazil; ^2^ Centro de Biologia Molecular e Engenharia Genética, Universidade Estadual de Campinas, Campinas, Brazil; ^3^ Institute of Cytology and Genetics, Siberian Branch of the Russian Academy of Sciences, Novosibirsk, Russia; ^4^ Frontier Engineering School, Novosibirsk State University, Novosibirsk, Russia; ^5^ Embrapa Agricultura Digital, Campinas, Brazil; ^6^ Department of Plant Biotechnology and Bioinformatics, Ghent University, Ghent, Belgium; ^7^ VIB Center for Plant Systems Biology, Ghent, Belgium; ^8^ Departamento de Genética, Evolução, Microbiologia e Imunologia, Instituto de Biologia, Universidade Estadual de Campinas, Campinas, Brazil

**Keywords:** *Agrobacterium*, B104, *BABY BOOM*, CRISPR/Cas9, off-target, protoplast, *VIRESCENT YELLOW-LIKE*, *WUSCHEL*

## Abstract

The recalcitrance exhibited by many maize (*Zea mays*) genotypes to traditional genetic transformation protocols poses a significant challenge to the large-scale application of genome editing (GE) in this major crop species. Although a few maize genotypes are widely used for genetic transformation, they prove unsuitable for agronomic tests in field trials or commercial applications. This challenge is exacerbated by the predominance of transformable maize lines adapted to temperate geographies, despite a considerable proportion of maize production occurring in the tropics. Ectopic expression of morphogenic regulators (MRs) stands out as a promising approach to overcome low efficiency and genotype dependency, aiming to achieve ’universal’ transformation and GE capabilities in maize. Here, we report the successful GE of agronomically relevant tropical maize lines using a MR-based, *Agrobacterium*-mediated transformation protocol previously optimized for the B104 temperate inbred line. To this end, we used a CRISPR/Cas9-based construct aiming at the knockout of the *VIRESCENT YELLOW-LIKE (VYL)* gene, which results in an easily recognizable phenotype. Mutations at *VYL* were verified in protoplasts prepared from B104 and three tropical lines, regardless of the presence of a single nucleotide polymorphism (SNP) at the seed region of the *VYL* target site in two of the tropical lines. Three out of five tropical lines were amenable to transformation, with efficiencies reaching up to 6.63%. Remarkably, 97% of the recovered events presented indels at the target site, which were inherited by the next generation. We observed off-target activity of the CRISPR/Cas9-based construct towards the *VYL* paralog *VYL-MODIFIER*, which could be partly due to the expression of the *WUSCHEL (WUS)* MR. Our results demonstrate efficient GE of relevant tropical maize lines, expanding the current availability of GE-amenable genotypes of this major crop.

## 1 Introduction

Maize (*Zea mays* L.) is the most produced grain globally because of its extensive and diverse utilization as food, feed, fuel and industrial raw material (Andorf et al., 2019), with United States, China, Brazil and Argentina currently being the four largest producers (Erenstein et al., 2022). Approximately 30% of the global maize production occurs in tropical geographies that have benefited from greater rates of annual yield gain than those of temperate areas, yet tropical yields are roughly half of those attained in the temperate zone (Edmeades et al., 2017; Erenstein et al., 2022). In addition to limitations such as poor soil fertility, pest incidence and extensive rainfed cultivation, maize yields in the tropics are also adversely affected by a shorter breeding history compared to their temperate counterparts (Edmeades et al., 2017).

Adopting genetically modified organisms (GMOs) in agriculture is an important contributor towards several of the United Nations’ 2015 Sustainable Development Goals by 2030 (Adenle et al., 2020). However, while considering the current consumer and regulatory concerns and consequently that deregulating a GMO product is a lengthy and expensive process, it is unlikely that breeding (both traditional and marker-assisted) can keep pace with the increasing global demand for higher and more stable crop yields. Emerging technologies such as genome editing (GE) could alleviate biosafety concerns while making biotechnological solutions more affordable (Chen et al., 2019; Ahmad, et al., 2021a; Gao, 2021). Importantly, these may synergize with breeding and transgenesis to accelerate the development of improved crops to feed the human population expected to reach 10 billion people by 2050, an important concern aggravated by the rapidly changing global climate and continuous decrease in arable land availability (Ahmar et al., 2020; Ahmad et al., 2021b; Massel et al., 2021).

CRISPR/Cas9-based targeted mutagenesis has been shown to be a promising tool for GE in maize, allowing manipulation of genes underlying agronomic traits, including complex traits controlled by multiple genes (Hernandes-Lopes et al., 2023). However, bringing such approaches to a commercial scale is difficult. Transformation recalcitrance of most maize genotypes, from temperate and tropical origins, is the main bottleneck for applying biotechnological solutions to this crop. In addition, most GE studies in maize are limited to temperate genotypes suitable for *Agrobacterium tumefaciens*-mediated transformation, such as the public inbred line B104 (Kausch et al., 2021; Yassitepe et al., 2021; Hernandes-Lopes et al., 2023). Therefore, the lack of genotypes both adapted to different agroecological zones and amenable to GE technologies is a significant limitation to maize improvement efforts in tropical and subtropical regions, which encompass developing countries most impacted by global climate change (Anderson et al., 2020 and references therein).

Given the importance of bringing transgenic and GE approaches into agronomically relevant maize cultivars, a variety of genotype-independent transformation methods has been developed in recent years. These methods often address two critical processes required for plant transformation: susceptibility to *Agrobacterium* infection and tissue competence for plant regeneration. The former can be overcome by the employment of ternary vector systems in which a helper plasmid harbors several *VIR* genes, which are able to greatly increase *Agrobacterium* infection of the explants (Anand et al., 2018; Zhang et al., 2019). On the other hand, plant regeneration has been improved by the ectopic overexpression of plant transcription factors known as morphogenic regulators (MRs): *BABY BOOM* (*BBM*), *WUSCHEL2* (*WUS2*) and *GROWTH REGULATORY FACTOR/GRF INTERACTING FACTOR* (*GRF*/*GIF*) (Lowe et al., 2016; Debernardi et al., 2020). These genes promote somatic embryogenesis or regeneration of shoots, improving the efficiency of plant transformation. This approach has been successfully used to transform different crops, such as cotton, rice, soybean, wheat, and maize, including genotypes otherwise recalcitrant to *Agrobacterium*-mediated transformation (Lowe et al., 2016; 2018; Zhang et al., 2019; Debernardi et al., 2020; Hoerster et al., 2020; Masters et al., 2020; Aesaert et al., 2022; Che et al., 2022).

Recently, a significant increase in transformation efficiency of the maize inbred line B104 was achieved by combining the expression of *BBM* and *WUS* with improved tissue culture media (Aesaert et al., 2022). In this approach, the use of constructs in which morphogenic genes are flanked by a developmentally controlled Cre/LoxP recombination system and the selection maker for resistance to imazapyr (*Highly Resistant ALS*; *HRA*) led to the generation of events with reduced T-DNA copy number and fertile T_0_ plants, while increasing transformation efficiency from 1% to 5%. The addition of a Cas9/sgRNA cassette in the construct has confirmed the functionality for gene editing applications, as exemplified by the knockout of the *VIRESCENT YELLOW-LIKE* (*VYL*) gene. This gene can be conveniently used as an early visual indicator for GE, as *vyl* loss-of-function leads to impaired stacking of chloroplast thylakoids, resulting in chlorotic pale-yellow leaves (Xing et al., 2014).

Despite the growing number of publications on improved maize transformation and GE technologies, successful reports on tropical maize lines have been less numerous than those on their temperate counterparts (Rangari et al., 2023; Souza et al., 2017; reviewed by Hernandes-Lopes et al., 2023; Yadava et al., 2017). Thus, in the present study, we applied the MR-based approach previously used by Aesaert et al. (2022) for optimized B104 transformation and GE to different tropical maize lines. Considering that maize transformation with *Agrobacterium* using immature zygotic embryos (IZEs) as explants is known to be highly ear-to-ear variable and affected by donor plant vigor and embryo competence in an environment-dependent manner (Ishida et al., 2007; Cho et al., 2015; Kausch et al., 2021; Aesaert et al., 2022), we carried out an initial experiment to evaluate the efficacy of such MR-mediated transformation protocol in B104 plants grown in a local greenhouse under tropical climate conditions in Campinas, São Paulo state, Brazil. Upon succeeding in establishing the MR-based genetic transformation and GE technology for B104 under these conditions, we next successfully established a GE platform for agronomically valuable tropical lines, including publicly available ones from the International Maize and Wheat Improvement Center (CIMMYT) as well a proprietary line from a Brazilian commercial maize breeding program.

## 2 Materials and methods

### 2.1 Plant material

Tropical maize inbred lines selected for genetic transformation and GE include three public lines from CIMMYT (CML360, CML444 and CML488) and two proprietary commercial lines (hereafter referred to as PCL1 and PCL2) from SEMPRE AgTech/WIN (https://sempre.agr.br/win), a Brazilian maize seed company. The tropical CMLs were developed at different CIMMYT Global Maize Program breeding sites and are adapted to some of the tropical/subtropical environments targeted by CIMMYT and partner institutions in South America (CML360) and Africa (mid-altitude/subtropical lowland; CML444 and CML488) (CIMMYT Global Maize Program, 2015). CML seeds are freely available and represent an important genetic resource for maize breeding and genetic studies (https://www.cimmyt.org/resources/seed-request/). In addition to the five tropical lines, we also used the temperate model inbred line B104 as a standard, since we sought to test the improved methods described by Aesaert et al. (2022) focused on this line, which in addition is the model line for numerous reports of successful transformation and GE.

B104 and CML plants used as source of explants were grown in a greenhouse at the Centro de Biologia Molecular e Engenharia Genética, Universidade Estadual de Campinas (Campinas, São Paulo state, Brazil), in 10 L pots filled with a commercial soil mix (Biogrow, Agrolink, Brazil) and vermiculite (at 4:1 proportion) supplemented with fertilizers. Supplemental light (Osram Zelion HL300, providing an additional 400 μmol m^−2^ s^−1^) was used to reach a 14-h light: 10-h dark photoperiod at 20°C–28°C. Ears were collected for embryo extraction between 12 and 16 days after pollination (DAP) during the months of May and December 2021, as well as in April and November 2022. PCL plants were grown in the field at Santa Helena de Goiás, Goiás state, Brazil, and ears were harvested at 13 DAP in February 2022, packed in Styrofoam boxes, and shipped to Campinas at 4°C.

### 2.2 Vector and gene construct

The vector used for GE experiments (pLAPAU17-VYL) was previously described in Aesaert et al. (2022). In summary, the construct’s T-DNA comprises the CRISPR machinery (Cas9 and sgRNA), the HRA selective marker for resistance to imazapyr, and a *BBM/WUS* expression cassette flanked by LoxP sites for excision of the morphogenic regulators. The *CRE* recombinase gene is also present between the LoxP sites, and its expression is driven by the pZmGLB1 promoter from maize *GLOBULIN-1*, which is active in the late-embryogenesis stage. Finally, the construct harbors a mRuby fluorescent marker which is active only when the MR expression cassette is excised from the T-DNA, bringing together the mRuby coding sequence and an *EF1α* promoter (pBdEF1α) (Supplementary Figure S1A). A GFP-expressing vector (pGC69) was used to assess protoplast transfection efficiency. This vector harbors a GFP coding sequence (containing an intron to avoid expression by *Agrobacterium*) under the control of a maize ubiquitin promoter (*pZmUBI*) (Supplementary Figure S1B). The plasmid was synthesized by DNA Cloning Service (https://dna-cloning.com/) and the sequence of its T-DNA is provided in Supplementary Table S1. The vector UBQ:RUBY (Addgene #160909) was used for evaluating IZEs susceptibility to *Agrobacterium* infection. The RUBY construct causes the conversion of tyrosine into the red pigment betalain (He et al., 2020) and was used as an early visual marker of transient expression following the genetic transformation procedure.

### 2.3 Prevalidation of GE activity in tropical maize protoplasts

Mesophyll protoplasts were isolated from leaves of etiolated seedlings grown in the dark for 6–10 days at 25°C. The protoplast isolation procedure was based on the protocol described by Shan et al. (2014). Leaves were cut into thin strips, incubated in 0.5M D-sorbitol plasmolysis solution, and cell walls were digested for 6 h with MacerozymeR-10 and cellulase R-10 (Duchefa Biochemie B.V, Haarlem, Netherlands). After washing and pelleting, protoplasts were co-transfected with the mixture of pGC69 and pLAPAU17-VYL vectors in three replicates per plant genotype. The transfection and further analysis were performed according to Gerasimova et al. (2019). After transfection, protoplasts were incubated for 2 days in the dark at room temperature. Then, the samples were evaluated under the microscope (Carl Zeiss™ Axio Vert. A1, Zeiss Filter Set 38HE) for estimating the ratio of GFP-expressing cells. Genomic DNA (gDNA) was isolated from protoplasts, and regions of *VYL* (also known as *Chr.9_ClpP5*; Zm00007a00050679 in B104) and its paralog *VYL-MODIFIER* (also known as *Chr.1_ClpP5*; Zm00007a00035036 in B104) (Xing et al., 2014) containing the target and off-target motifs, respectively, were amplified using nested PCR with two primer pairs for each gene (Supplementary Table S2). Laboratório Central de Tecnologias de Alto Desempenho em Ciências da Vida (LACTAD), a service facility at the Universidade de Campinas, performed library preparation and deep amplicon sequencing. Mutation pattern and frequency were evaluated for pooled libraries of three biological replicates using the Small Indel Analyzer (SIA) script (https://github.com/vikhall/Small_Indell_Analyzer). The frequency of mutations was normalized according to the transfection efficiency by dividing the raw mutation frequency by the average ratio of GFP-expressing cells.

### 2.4 Plant transformation

Immature zygotic embryos (IZEs) were submitted to *Agrobacterium*-mediated transformation as previously described by Coussens et al. (2012) and Aesaert et al. (2022). Briefly, IZEs were obtained by fertilizing ears with pollen from sibling plants. IZEs ranging from 1.5 to 2 mm long (12-16 DAP) were carefully excised from previously sterilized ears using a sterile scalpel and micro-spatula. For infection, *Agrobacterium* strain EHA105 harboring pLAPAU17-VYL vector was inoculated in infection medium containing acetosyringone (100 µM) and maintained in a shaker at 28°C, 150 rpm. After 2 h, the bacterial culture was adjusted to an optical density of 0.3–0.4 and used for IZEs infection by co-cultivation at room temperature in the dark for 5 min. IZEs were plated with the scutellum side up on co-cultivation medium at 21°C for 3 days in the dark. Following co-cultivation, IZEs were transferred to a resting medium without Imazapyr selection and incubated at 25°C in the dark for 6 days. Subsequently, IZEs were exposed to a selection medium containing Imazapyr for 7 days at 25°C in the dark. In the next phase, IZEs were subjected to a two-step maturation process: first, for 14 days in the dark on a medium containing cupric sulfate (CuSO_4_), Indoleacetic-acid (IAA), thidiazuron, abscisic acid (ABA), 6-benzylaminopurine (BAP) and zeatin, followed by 14 days under light on a medium containing CuSO_4_, IAA, ABA, and BAP. The final stage involved transferring plantlets to regeneration II medium and maintaining them for 14 days at 25°C under light. Following tissue culture, plantlets with well-developed roots were transferred to horticultural peat pellets (Jiffy-7) and soil mix and maintained in a controlled growth room at 25°C, 110  μmol m^-2^ s^−1^ and 16-h light: 8-h dark photoperiod. After 30 days, plants were transferred to 10 L pots and grown in the greenhouse. The material preparation, culture media and sterilization process are described in Coussens et al. (2012) and Aesaert et al. (2022). A small proportion of IZEs (ten to 25 per ear) was transformed with UBQ:RUBY to determine *Agrobacterium* susceptibility. IZEs transformed with UBQ:RUBY were inspected for red color 7 days after transformation. Different stages of explant development were photographically documented using a Leica M165 FC stereomicroscope. mRuby fluorescence was observed using a mCherry - M205FA/M165FC filter, 3 days after *Agrobacterium* transfection.

### 2.5 Plant genotyping

Leaf samples weighing approximately 50 mg were collected into 1.5 mL tubes containing five 2 mm ceramic beads and immediately frozen in liquid nitrogen. The material was homogenized using a PowerLyzer 24 Homogenizer (Qiagen) and used for gDNA isolation using the Wizard^®^ Genomic DNA Purification kit (Promega). The gDNA samples were used as templates for PCR amplification of different regions of the T-DNA, including 1) the sgRNA, 2) the MR gene *WUS*, and 3) the region comprising the entire MR cassette (∼10.6 kb), which should only yield a PCR product in the case of its successful excision, resulting in a smaller 1,202-bp amplicon (see Supplementary Figure S1A). Additionally, gDNA samples were used for amplification of the *VYL* and *VYL-MODIFIER* genes (Xing et al., 2014). PCRs were performed using the GoTaq^®^ G2 DNA Polymerase (Promega) according to the manufacturer’s instructions. For all PCRs, the same cycling program was used: initial denaturation at 95°C for 2 min, followed by 35 cycles at 95°C (30 s), 57°C (45 s) and 72°C (45 s except for the analysis of the MR cassette excision, in which case 1 min and 30 s was used).

The resulting amplicons were purified using the Wizard^®^ SV Gel and PCR Clean-Up kit (Promega) and submitted to Sanger sequencing at the LaCTAD facility. Sequence data were analyzed using the ICE tool (Synthego) (https://ice.synthego.com/). A list of all primers used in this study can be found in Supplementary Table S2. Zygosity analysis of T_0_ and T_1_ plants was based on ICE results following a modification of Pan et al. (2022) criteria as follow: (i) WT, total mutation frequency <15%; (ii) homozygous, one type of mutation ≥70%; (iii) heterozygous, one type of mutation ≥30% and total mutation frequency <70%; (iv) biallelic, total mutation frequency ≥70% with two major types of indels presenting ≥30% each. Any remaining cases were classified as mosaic.

Additionally, DNA samples from 16 T_0_ plants (including 15 mutants and one WT) with ICE scores (*R* ^2^) of at least 0.95 were taken for NGS analysis. Deep amplicon sequencing and data processing were performed according to the methodology applied in the protoplast experiment. The NGS analysis of mosaic T_0_ plants was performed to compare ICE and SIA tool results and experimentally validate the latter for the *VYL* target site. The statistical significance of the correlation of indel frequencies obtained with SIA and ICE was calculated using the ggscatter function from the ggbubr package in R. The Pearson correlation coefficient was used.

### 2.6 Phenotyping of T_1_ plants

T_1_ seeds (16 per line) were sown into seedling tubes filled with soil mixture and watered daily. Nine days after sowing, plants were photographed and analyzed for *VYL* and *VYL-MODIFIER* loss-of-function phenotypes, which were categorized into WT, *vyl* and albino, based on the leaf color patterns.

## 3 Results

### 3.1 Genome editing validation in maize protoplasts

**FIGURE 1:**
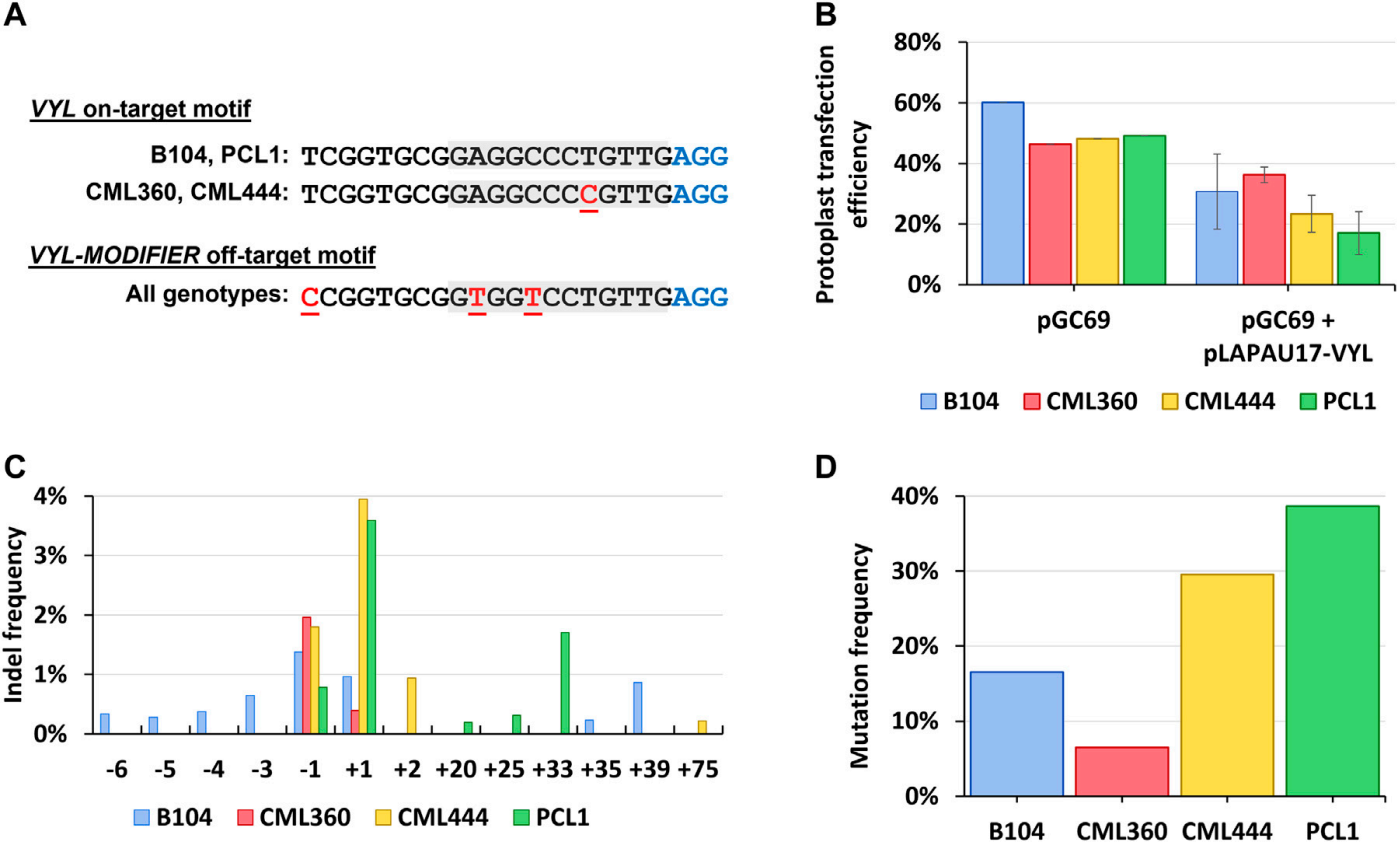
**(A)** *VYL* (*Chr.9_ClpP5*) and *VYL-MODIFIER* (*Chr.1_ClpP5*) target and off-target sequences, respectively, in different maize lines. Red, underlined letters: mismatches compared to the sgRNA spacer sequence; gray boxes: spacer “seed” region; blue “AGG”: PAM site. **(B)** Protoplast transfection efficiency counted as the proportion of GFP positive cells. **(C)** Assessment of indel frequencies in leaf-derived protoplasts transfected with pLAPAU17-VYL. **(D)** Mutation frequency counted as the proportion of mutated to WT reads normalized by the transformation efficiency of corresponding samples.

Considering that two of the tropical genotypes (CML360 and CML444) have a SNP (C to T) in the target motif of the *VYL* gene, five nucleotides upstream of the PAM sequence (Figure 1A), we assessed the construct’s efficacy to induce indels at the target site in the different maize lines using a maize protoplast system prior to scaling up the plant transformation experiments. Mesophyll protoplasts from B104, CML360, CML444, and PCL1 lines were prepared, co-transfected with the plasmid vector pGC69 (GFP-expressing) together with the pLAPAU17-VYL construct, and then evaluated for mutation patterns and frequency by employing deep sequencing of amplicons containing the *VYL* target region. We used the same strategy to assess the known off-target activity of this construct in inducing indels at the *VYL* paralog *VYL-MODIFIER* gene, which contains three mismatches at the sgRNA targeting sequence (Figure 1A).

The protoplast transfection efficiency reached 46%–60% for all lines when using pGC69 alone (Figure 1B; Supplementary Figure S2), and 17%–36% when co-transfected with pGC69 and pLAPAU17-VYL (Figure 1B). The mutagenesis efficiency in protoplasts showed variability across different genotypes. Remarkably, the pLAPAU17-VYL construct was capable of inducing mutations in tropical lines carrying a mismatched VYL allele (CML360 and CML444), albeit with a lower efficiency in CML360 (Figures 1C, D).

Notably, the two lines with a perfect match between the sgRNA and the target sequence (B104 and PCL1) presented greater diversity of induced mutations compared to the lines with the mismatched allele (Figure 1C). We verified that the predominant indel types found in CML360 and CML444 were single nucleotide insertions and single nucleotide deletions, while PCL1 also presented long insertions, and B104 contained either single nucleotide indels or long insertions and deletions (Figure 1C). All identified long insertions consisted of sequences originating from different regions of the vector backbone. On the other hand, the analysis of *VYL-MODIFIER* did not reveal mutations in most of the samples, with a unique exception for one of the PCL1 replicates, for which a restricted range of mutation types was noticed.

Considering that the *VYL*-targeting construct showed activity in all maize genotypes, despite the presence of the C to T single mismatch at the target site in CML360 and CML444, and that the construct activity was mostly specific towards the *VYL* gene, we proceeded to plant stable transformation experiments using the pLAPAU17-VYL construct.

### 3.2 Transformation of B104 and tropical lines

**FIGURE 2:**
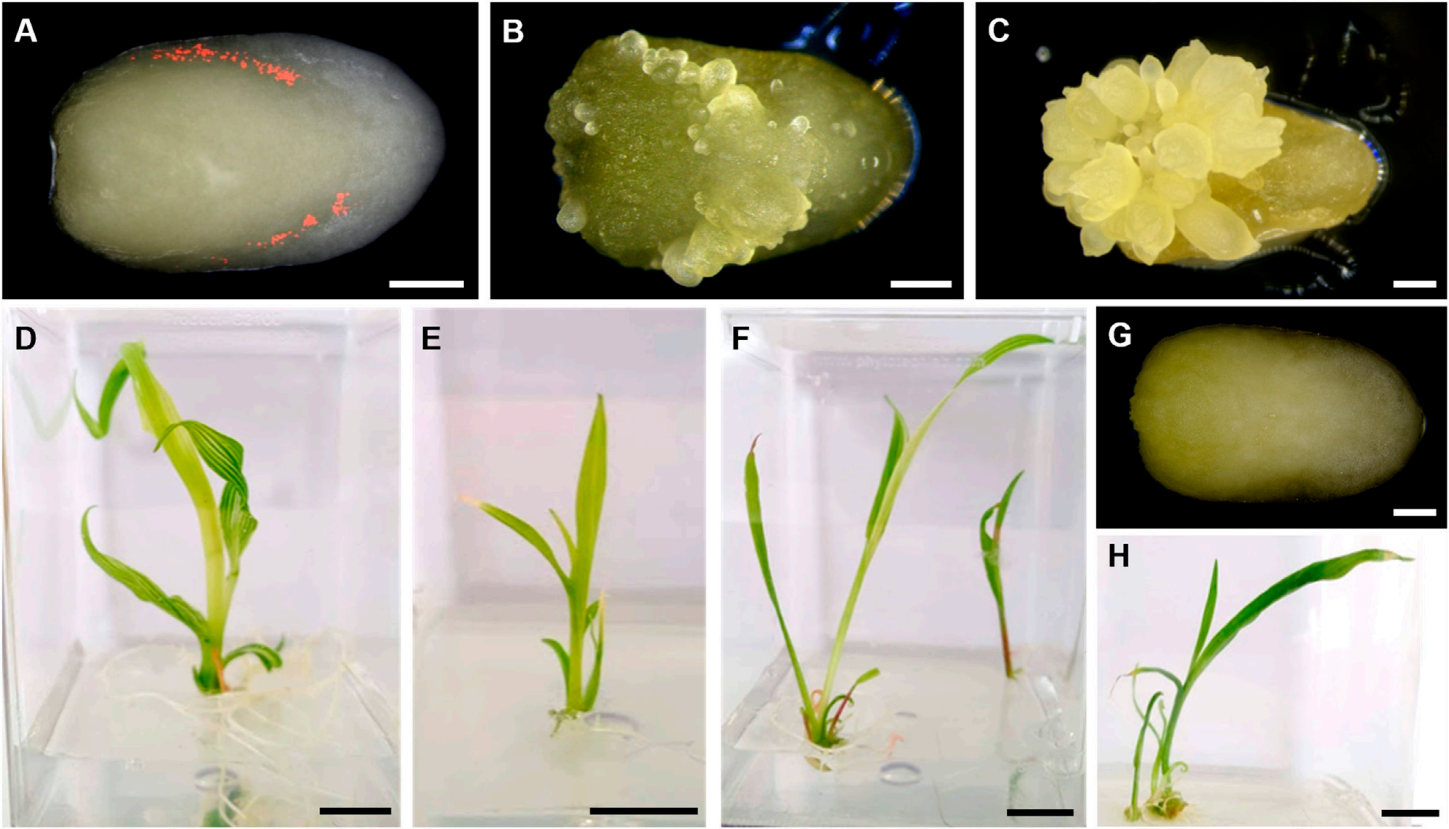
Explants (immature zygotic embryos–IZEs) and regenerant plants of maize B104 inbred line. **(A)** Transient expression of mRuby, 3 days post infection. **(B)** Initiation of somatic embryos observed 8 days post infection. **(C)** Somatic embryos developing at the surface of the immature embryo 17 days post infection. **(D–F)** Regenerant plants showing the pale-yellow *vyl* loss-of-function phenotype. **(G)** Control embryo 3 days post infection. **(H)** WT plant. White scale bar = 1 mm; Black scale bar = 1 cm.

Since B104 is a temperate line not adapted for optimal growth in tropical regions, donor plant vigor could negatively impact embryo competence to *Agrobacterium*-mediated transformation, consequently reducing its efficiency. Considering this, we performed an experiment to evaluate the efficacy of the MR-mediated transformation protocol in B104 plants grown in Campinas’ tropical climate conditions. Three days post *Agrobacterium* infection of IZEs, transient expression of mRuby reporter gene was observable in scutellum spots (Figure 2A), indicating excision of the MR expression cassette. Eight days post infection, initiation of somatic embryos could be seen on the surface of the scutellum (Figure 2B), and its development proceeded normally (Figure 2C), resulting in healthy plantlets (Figures 2D–F). From 1,537 transformed B104 IZEs, we recovered 31 regenerants, all of which presented integration of the T-DNA as detected by PCR, indicating an overall transformation efficiency of 2.02% (Table 1). Notably, transformation efficiency was highly variable between different ears. Only three out of eight ears used as sources of explants yielded transgenic events, with 0.78%, 9.92% and 10.96% transformation efficiencies each (Supplementary Table S3). This is a significant effect often observed in maize transformation, but not well addressed thus far. All plants presented the expected pale-yellow *vyl* phenotype while still growing *in vitro* (compare Figures 2D–F,H). From these, 13 plants were grown to maturity and crossed with WT B104. Except for one event, all crosses yielded seeds.

**TABLE 1:** Summary of transformation experiments with maize inbred line B104 and tropical maize lines.

Genotype	Number of ears	Embryo age^a^	Transformation date	Number of embryos	Regenerant plants	Transformation efficiency^b^
B104	8	16	05/05/2021	1,537	31	2.02%
CML360	5	12	05/04/2022	913	5	0.55%
CML360	6	15	11/11/2022	776	6	0.77%
CML444	1	14	15/12/2021	191	10	5.24%
CML444	2	12	18/12/2021	341	8	2.35%
CML488	2	12	18/12/2021	167	0	0.00%
CML488	7	12	12/04/2022	717	0	0.00%
PCL1	3	13	25/02/2022	181	12	6.63%
PCL2	4	13	25/02/2022	218	0	0.00%

^a^ Days from pollination to harvest.

^b^ Number of recovered transgenic plants/total number of initial embryos.

**FIGURE 3:**
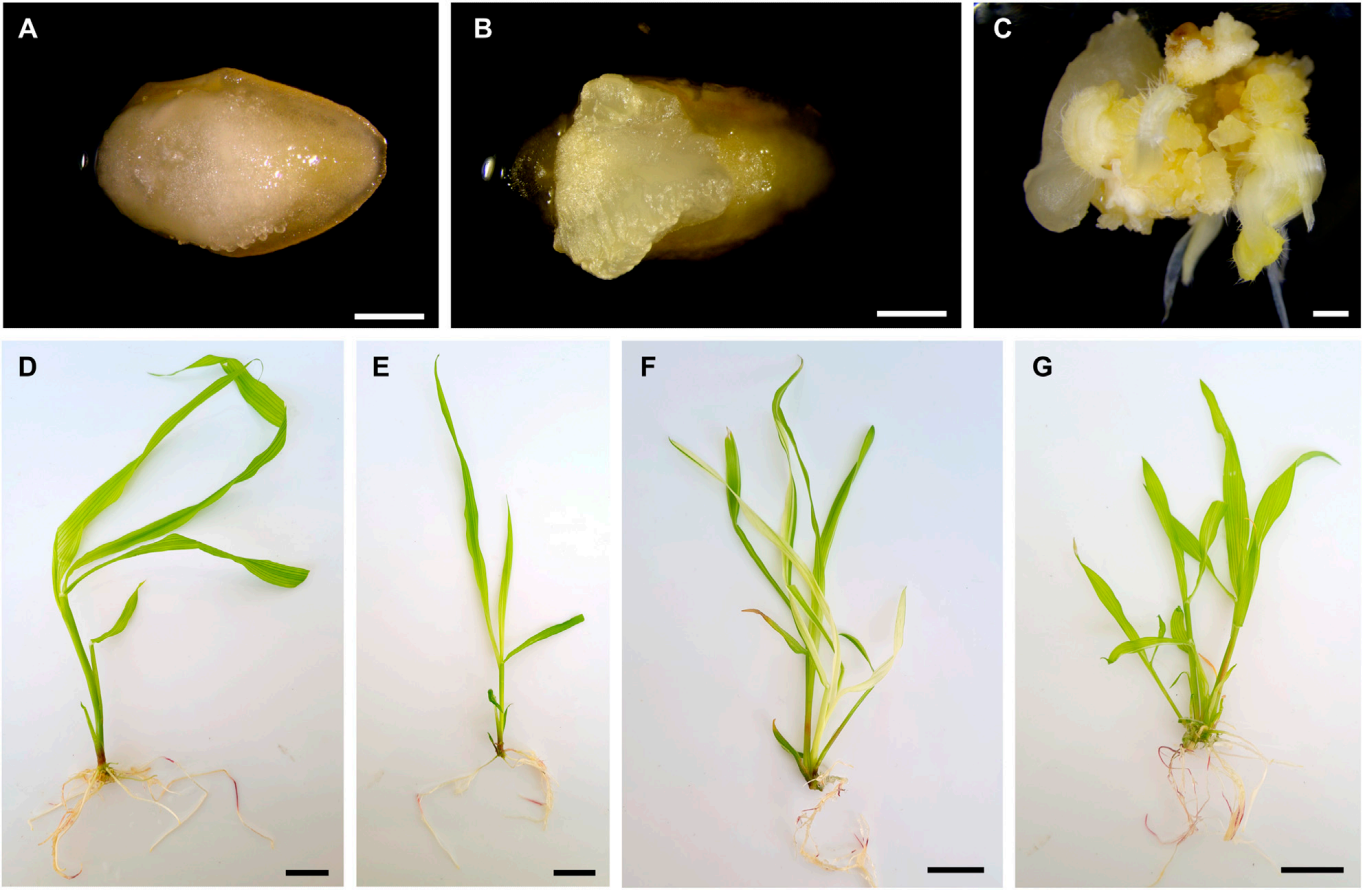
Explants and regenerant plants of tropical maize lines. **(A–C)** IZEs of CML360. **(A)** Initiation of somatic embryos observed 9 days post infection. **(B)** Callus structure forming on the scutellum of an IZE, 15 days post infection. **(C)** Callus showing initiation of organogenesis. **(D–G)** Regenerant plants of CML444 showing the pale-yellow *vyl* loss-of-function phenotype. White scale bar = 1 mm; Black scale bar = 2 cm.

Next, the same transformation protocol was applied to five tropical maize genotypes, from which three (CML360, CML444 and PCL1) were amenable to transformation using the *BBM*/*WUS* morphogenic regulators, while CML488 and PCL2 have not yielded any regenerants (Table 1). As observed for B104, the somatic embryos initiated on the scutellum of IZEs (Figure 3A). However, explants from CML360 and CML444 developed differently. After the initiation of the somatic embryos, the IZEs formed an amorphous structure (Figure 3B), which subsequently developed into a callus usually observed in classical maize transformation protocols (Figure 3C). Regardless of these differences in explant development, CML360, CML444 and PCL1 yielded healthy transgenic plantlets (Figures 3D–G). Notably, in addition to not yielding regenerant plants, none of the embryos from CML488 and PCL2 transformed with UBQ:RUBY exhibited the expected red pigmentation typically observed during transient expression of this construct, suggesting that recalcitrance of such lines may rely on their resistance to *Agrobacterium* infection (Supplementary Table S4; Supplementary Figure S3).

In our experiments, CML444 and PCL1 were the most responsive lines to stable genetic transformation, with 3.38% and 6.63% overall transformation efficiency, respectively (Table 1, Supplementary Table S3). As observed in B104, lines amenable to transformation had a great variation in transformation efficiencies between different ears and different transformation experiments (Supplementary Table S3). While still young, most tropical regenerated plantlets exhibited the *vyl* phenotype (Figures 3D–G). Also, all regenerated plants were PCR positive for integration of the T-DNA, except three from CML360 and one from CML444, indicating the effectiveness of the selective marker. Finally, some CML360 and PCL1 T_0_ events were grown to maturity and either selfed or crossed with WT B104 to produce seeds.

### 3.3 Genotyping of transgenic events and genome editing efficiency

**FIGURE 4:**
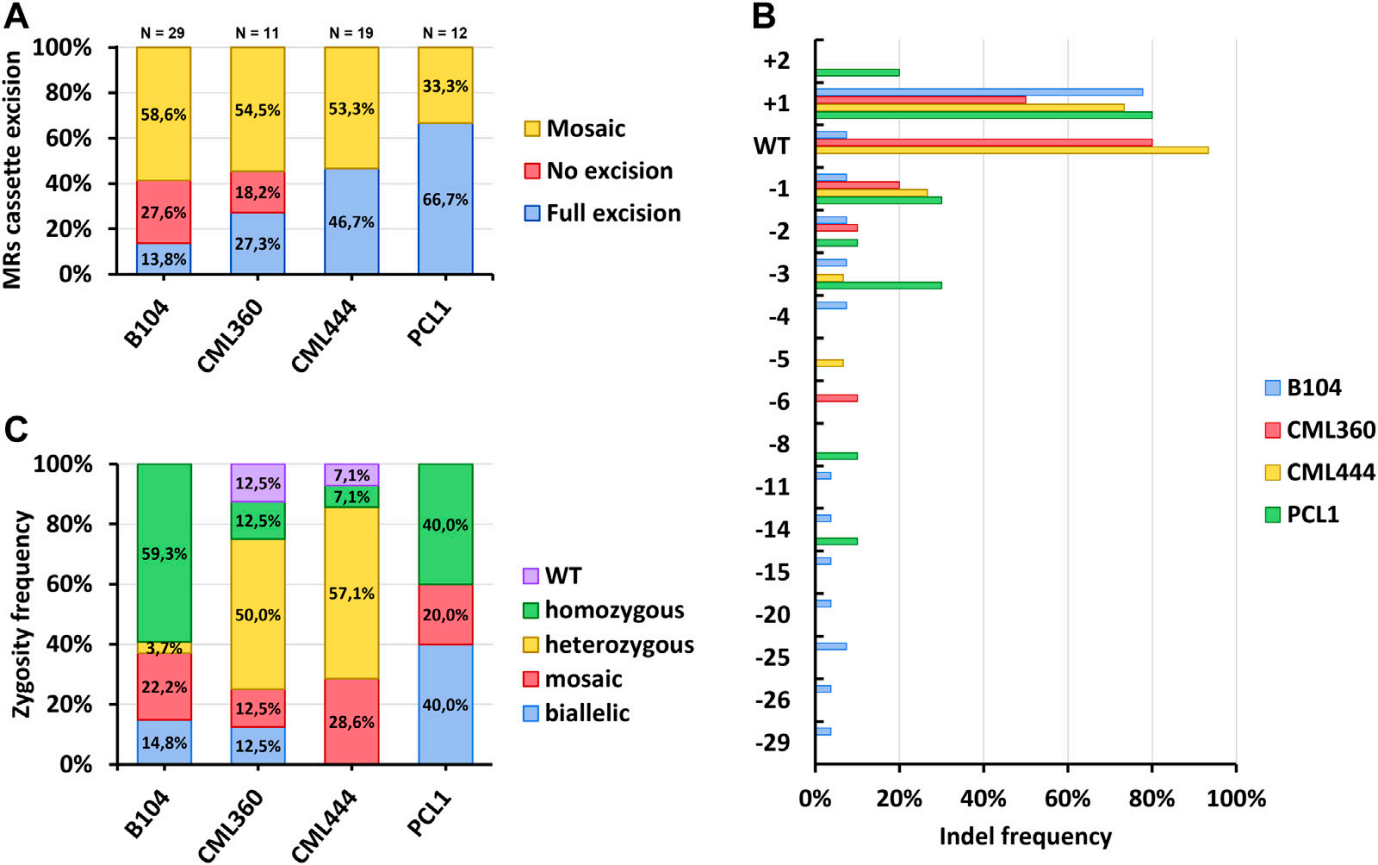
Genotyping of T_0_ events. **(A)** Frequency of excision of the morphogenic genes expression cassette. **(B)** Frequency of T_0_ events harboring diverse indel types. **(C)** Zygosity analysis of the *VYL* locus in T_0_ events.

After acclimation, gDNA was isolated from leaf samples of all T_0_ individuals for genotyping. We first performed multiple PCRs to verify the presence of the T-DNA, as well as to check whether the MR expression cassette was indeed excised by the CRE/LoxP recombination system (Supplementary Figure S4). As previously mentioned, nearly all recovered events presented integration of the T-DNA. However, excision of the MRs cassette was highly variable. For B104, only three out of the 29 tested plants were positive for the excision and negative for the presence of the MR cassette, corresponding to 13.8%; whereas eight (27.6%) of the plants were only positive for the presence of the MRs cassette. The remaining 17 (58.6%) plants were mosaics (i.e., PCRs for both presence and excision of the MRs were positive; Figure 4A, Supplementary Table S5). Notably, the developmental-induced Cre/Lox system was more efficient in tropical lines, with full excision of the MRs cassette reaching up to 66.7% in PCL1 (Figure 4A). A single event of CML444 was negative for both presence and excision of the MRs cassette. This event was PCR positive for the sgRNA transgene, suggesting a partial integration of the T-DNA (Supplementary Table S5). Despite the relatively high proportion of plants still harboring the MRs cassette, all regenerated plants developed normally, including those in which the excision of the MRs was not complete.

Sanger sequencing followed by ICE analysis of the target site was performed to assess on-target editing efficiency. All sequenced plants from the B104 inbred line were edited at the target site, with only a low proportion of the WT allele present in a few samples (<10% in three events, 26% in a mosaic event, and 52% in heterozygous events; Supplementary Table S5). However, many CML360 and CML444 events retained the WT allele (Figure 4B; Supplementary Table S5). The most common induced indel was a single nucleotide insertion (“+1”). The frequency of T_0_ events harboring this indel ranged from 50% in CML360 to 80% in PCL1 (Figure 4B). Sequencing also allowed the identification of independent GE events originating from the same explant. For example, B104 had seven explants giving rise to two or three regenerants. From these, only one explant had all its corresponding regenerants presenting the same indel pattern (Supplementary Table S5). A similar scenario was found in the tropical lines, with most regenerants from a single explant presenting independent GE events, and only two cases of regenerants from the same explant with the same indel patterns (Supplementary Table S5).

The zygosity analysis of independent GE events unveiled distinct patterns in different maize lines. In B104, homozygous editing, characterized by a high frequency (>70%) of a single type of indel, predominated. Conversely, PCL1 exhibited a prevalence of both homozygous and biallelic events, which involves two major (>30%) indel types. Notably, CML360 and CML444 displayed a pronounced prevalence of heterozygous events, in which one major indel type was observed alongside the WT allele. Only these lines produced events in which the *VYL* allele was considered WT, specifically characterized by a WT allele frequency exceeding 90% in the ICE analysis. All lines exhibited mosaic GE events, evidenced by the presence of multiple indels with varying frequencies (Figure 4C, Supplementary Table S5). The ICE results were compared with the deep amplicon sequencing data for 16 T_0_ plants. Both approaches showed high correlation (R = 0.97, *p* < 2.2 × 10^−16^) and provided similar mutation frequencies (Supplementary Figure S5). The high correlation between the two datasets confirms the efficiency of SIA for *VYL* mutation detection starting from frequencies about 1% and higher. SIA was also applicable for frequencies lower than 1%, in a range where ICE was not capable of detecting mutations.

### 3.4 *In planta* off-target activity

**FIGURE 5:**
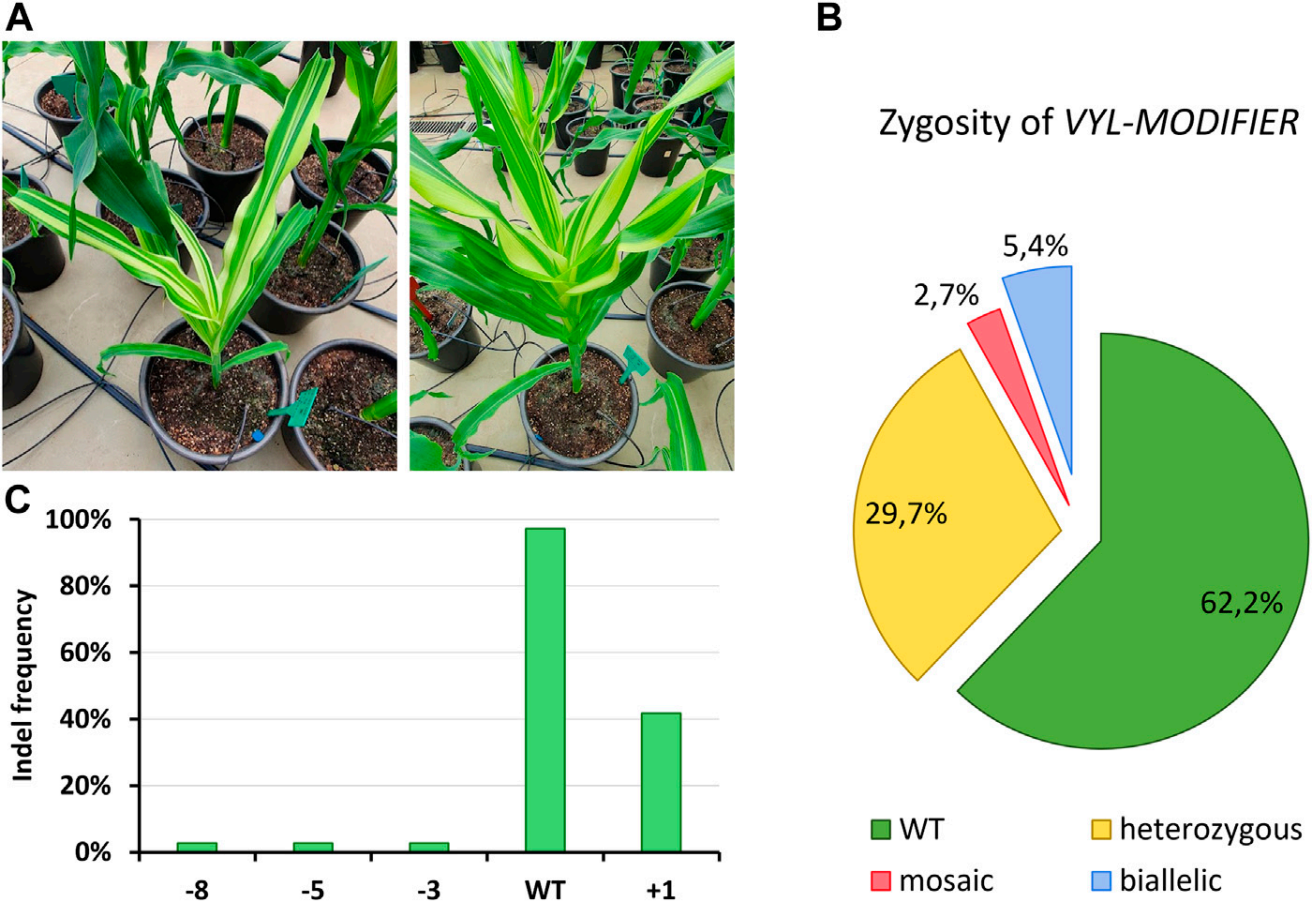
*VYL-MODIFIER* off-target editing in tropical maize lines. **(A)** T_0_ events showing leaves with mosaic albino stripes. The pot diameter is 30 cm. **(B)** Zygosity analysis of the edited *VYL-MODIFIER* locus in T_0_ events. **(C)** Frequency of tropical T_0_ events harboring diverse indel types.

Many of our T_0_ edited plants showed phenotypic signs of *VYL-MODIFIER* loss-of-function (i.e., leaves with albino stripes; Figure 5A) although our protoplast validation assay showed very low off-target activity. To analyze the off-target editing of *VYL-MODIFIER,* we performed Sanger sequencing followed by ICE analysis of this gene in the tropical lines. The sequencing results confirmed that 37.8% of the tropical T_0_ events also had indels at the *VYL-MODIFIER* off-target site (Figure 5B). Again, the insertion of a single nucleotide was the most common indel in the off-target region, present in 41.7% of the events (Figure 5C). Such mutations appear mostly as heterozygous events. No GE event was homozygous for the off-target mutation (Figure 5B).

### 3.5 Inheritance of indels and phenotypes

We randomly selected two independent T_0_ events of B104, CML360 and PCL1 to test whether the indels observed in T_0_ would be inherited by T_1_ plants. Nine days after sowing, samples of all plants were subjected to PCR analysis to detect the presence of the T-DNA. A chi-square test confirmed that the observed segregation of the transgene in the tested lines, which encompassed those derived from crosses with WT B104, as well as one line obtained through self-fertilization, agreed with the expected Mendelian ratio (*p*-value <0.05) (Table 2). Sequence analysis revealed that, except for a specific indel (−15 in a B104 event), all the observed indels in the T_0_ generation were inherited by the transgene-free (Cas negative) T_1_ plants. Furthermore, a few novel indels emerged in these T_1_ plants, suggesting a mosaic state in the parental T_0_ (Table 2).

**TABLE 2:** Genotyping of T_1_ plants derived from randomly selected T_0_ events of B104, CML360 and PCL1.

					Nr. of plants		Non-transgenic T_1_	
T_0_ line	T_0_ event	Major indels (T_0_)	Zygosity (T_0_)	Cross	Cas +	Cas -	Indels	Nr. of plants
B104	62GC102_VI_8a	−15 (53%)/+1 (44%)	biallelic	B104	7	9	0/+1	4
							−42/0	5
	62GC102_VI_2c	+1 (89%)	homozygous	B104	8	8	−51/0	5
							0/+1	3
CML360	3CML_III_1c	0 (32%)/+1 (52%)	heterozygous	B104	6	9	0/0	2
							0/+1	5
							−1/0	2
	3CML_III_3a	−2 (54%)/0 (34%)	heterozygous	B104	2	8	−2/0	8
PCL1	1PCL_II_1a.1	−3 (100%)	homozygous	selfing	11	5	−3	4
	1PCL_II_5a	−14 (52%)/+1 (45%)	biallelic	B104	5	11	−14/0	5
							−6/0	2
							0/+1	1
							−3/0	1

**FIGURE 6:**
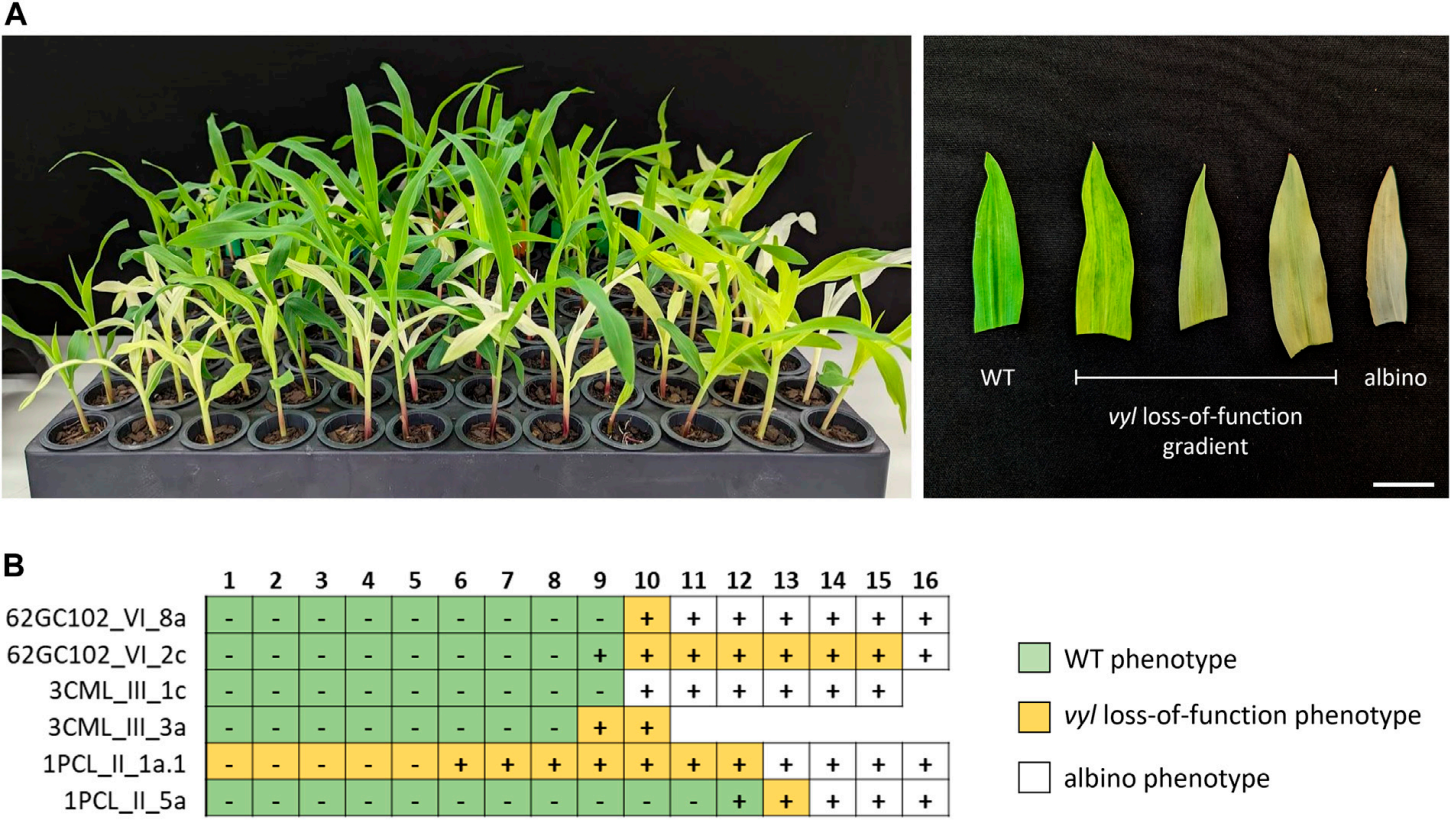
Analysis of T_1_ plants of two edited independent events of different maize lines. **(A)** Phenotypic gradient observed in seedlings (left) and leaves (right). **(B)** Genotyping of T_1_ plants, each row corresponding to an individual B104 (62GC1_VI), CML360 (3CML_III) or PCL1 (1PCL_II) T_0_ event. Boxes represent different individuals (1–16) in the T_1_ progeny. Plus and minus signs denote transgenic and non-transgenic individuals, respectively. Missing boxes represent seeds that failed to germinate. Scale bar = 2 cm.

All non-transgenic plants derived from backcrosses displayed the WT phenotype. In contrast, with the exception of two cases, plants retaining the CRISPR machinery exhibited a broad range of phenotypes, encompassing a gradient from the pale-yellow color, indicative of *vyl* loss-of-function mutations, to complete albino phenotypes, with variegations observed within these categories (Figures 6A, B), attributable to *vyl* and *vyl-modifier* double mutations. Accordingly, a diversity of new mutations was observed at the *VYL* and *VYL-MODIFIER loci* in transgenic plants, with all sequenced albino plants presenting some degree of indels at the *VYL-MODIFIER* gene. We also tested plants produced by self-pollination of a homozygous T_0_ GE (−3) event. In this case, no plant displayed the WT phenotype, including the non-transgenic ones.

## 4 Discussion

### 4.1 Using protoplasts to pre-screen sgRNAs efficiency

Protoplast methodologies are useful for diverse CRISPR/Cas-mediated GE techniques in plants. GE reagents (DNA, RNA, RNP) can be delivered into protoplasts via transfection to quickly assess the effectiveness of sgRNAs and Cas proteins (Lin et al., 2020; Sretenovic et al., 2021). In this study, we employed a protoplast test system based on analyzing cell populations obtained from pooling samples from a few dozen independent seedlings. This can give sufficient information on the target locus polymorphism in selected genotypes. The main question addressed with our protoplast experiments was whether the SNP present at the *VYL* target site in CML360 and CML444 (compared to our sgRNA) could affect mutagenesis efficiency. The SNP present in these two tropical lines is located at the seed region, five nucleotides upstream of the PAM site, which is expected to impair the Cas9 nuclease activity. The hypersensitivity to mismatches in the seed region of the target sequence has been previously documented in plants, as reported by Tang et al. (2018).

Our results showed that the presence of an SNP at the seed region of the *VYL* target site in the two CMLs was not sufficient to abolish the GE capability, although it had affected the mutagenesis pattern. While CML360 protoplasts exhibited the lowest normalized mutation frequency (6.5%) among the tested genotypes, CML444 demonstrated an almost twofold increase (29.5%) in mutation frequency compared to B104 (16.5%). When considering the mutation frequency solely, protoplasts results could not be directly translated into results found in plants, in which both CML360 and CML444 had a high proportion of the WT allele, indicating an overall less effective GE activity. However, protoplast and plant results were consistent regarding the sgRNA capability of inducing indels, as well as the indel diversity produced, which was much lower in the CMLs. Interestingly, the off-target activity was only observed in one protoplast sample out of twelve, which disagreed with the results observed *in planta*, in which case the off-target activity was detected in 37.8% of the tropical T_0_ events.

### 4.2 Tropical maize lines exhibit genotype-dependence to MR-mediated transformation

Achieving stable genetic transformation in maize poses a challenge that has historically been overcome only for a limited number of genotypes. While reports on the transformation amenability of tropical maize lines are comparatively scarce compared to their temperate counterparts (Yadava et al., 2017), studies have consistently shown a strong dependence on genotype for transformation efficiency. This genotype-dependency poses a significant hurdle in developing novel genetically modified varieties. Based on a recently published improved transformation protocol using *BBM*/*WUS* morphogenic regulators (Aesaert et al., 2022), we successfully established a GE platform for three out of five tested tropical lines, while also validating the use of the B104 line for transformation and GE under tropical conditions.

While the PCL1 line performed better in transformation efficiency (6.6%), it is important to note that we carried out a single transformation experiment with this line, using explants obtained from only three different ears. Since explant competence is highly variable between ears and experiments (Ishida et al., 2007; Cho et al., 2015; Kausch et al., 2021; Aesaert et al., 2022), a greater number of experiments is necessary to ascertain the amenability of this line to the protocol employed in this study. It is also important to point out that the embryos of this line were obtained from field-grown plants, which surprised us positively since environmental variations in growth and transport could skew the results. On the other hand, CML360 had the lowest transformation efficiency, observed in two independent experiments using a reasonable number of embryos (>700) harvested from five and six different ears. These experiments were also performed at different times of the year but resulting in comparable transformation efficiencies (0.55% and 0.77%). In addition, all ears of this line had a high percentage of IZEs transiently expressing the RUBY construct. Taken together, these results suggest that CML360 is consistently amenable to the transformation protocol, albeit with a low efficiency, which is probably related to explant regeneration rather than to a lack of *Agrobacterium* susceptibility.

Although we successfully transformed tropical maize lines using the *BBM/WUS* protocol, it is important to acknowledge that this is not a universal solution for the problem of maize recalcitrance to transformation. While maize transformation using immature embryos as explants has been shown to be highly dependent on the quality of the explants (Ishida et al., 2007; Cho et al., 2015; Kausch et al., 2021; Aesaert et al., 2022), we were unable to recover any transgenic events from multiple ears of CML488 and PCL2, indicating that these genotypes may not be amenable to the transformation protocol employed in this study. This outcome is not unexpected, as previous research has shown that some inbred lines may be recalcitrant to *BBM/WUS* transformation protocol. In fact, the seminal study that introduced the MR-based approach reported that only 33 out of 50 tested inbred lines could yield transgenic events (Lowe et al., 2016).

One important factor affecting maize transformation recalcitrance is IZE susceptibility to *Agrobacterium* infection. A “genotype-independent” transformation method was previously reported by combining the BBM/WUS approach with a ternary vector system, which facilitates this step (Lowe et al., 2018). A similar approach was successfully used for the transformation of the recalcitrant maize inbred line ND88 (Zhang et al., 2019). These ternary systems rely on equipping the *Agrobacterium* with a helper plasmid harboring additional *vir* genes, enhancing *Agrobacterium* infection. The employment of such a ternary vector system shows promise in addressing transformation recalcitrance in lines such as CML488 and PCL2, since both lines demonstrated the absence of transient expression of the RUBY construct in transformed IZEs, indicating that susceptibility likely plays a pivotal role in the observed transformation recalcitrance.

Taken together, these findings highlight that a combination of different strategies, including the optimization of *Agrobacterium*-mediated delivery, tissue culture conditions and the identification of novel morphogenic genes, such as *GRF-GIF* (Debernardi et al., 2020; Kong et al., 2020), will be necessary to overcome the maize recalcitrance to transformation and expand the range of amenable genotypes. Ultimately, genotypes not responsive to any of such strategies can, in principle, still be edited by alternative strategies such as the IMGE/Hi-Edit, which are based on GE coupled with haploid induction (Kelliher et al., 2019; Wang et al., 2019).

A recurrent challenge in using *BBM* and *WUS* as morphogenic genes is their pleiotropic effects, including developmental abnormalities and sterility resulting from their continuous expression. To mitigate these effects, the pLAPAU17 construct utilizes specific promoters to drive the expression of the MRs, while also harboring a self-excising Cre/LoxP system with the Cre recombinase driven by the ZmGLB1 promoter. In our study, complete excision of the MRs cassette was not efficient, being achieved in only a small proportion of transgenic events, with particularly low efficiency in B104. Thus, most of the regenerated plants displayed a mosaicism for the presence or absence of the MR genes, suggesting it happened at different stages of plant development. Despite the presence of the MR genes in many plants, no severe phenotype was associated with these transgenes. MR-positive, MR-negative and MR-mosaic plants were not phenotypically distinguishable. This observation is consistent both with the promoters used to drive MR expression, being specific for embryos and some vegetative tissues (Lowe et al., 2018). Also, variations in the successful full excision of the MRs cassette between the genotypes may indicate a stronger activity of the pZmGLB1 promoter in genotypes like CML444 and PCL1.

### 4.3 Highly efficient GE of tropical maize lines

GE of the target *VYL* locus was highly efficient as only two out of the 66 confirmed T_0_ events did not show any evidence of editing. This is in accordance with the data previously reported by Aesaert et al. (2022) using the same construct pLAPAU17-VYL. A remarkable observation in CML360 and CML444 was the high proportion of T_0_ events classified as heterozygous, i.e., events in which a WT allele was identified together with a mutated allele in frequencies close to 50% each. While this is consistent with the editing of only one allele, some plants are probably in fact mosaics, with approximately 50% of their cells edited at the target locus. This suggests a lower GE efficiency in both cases, probably related to the SNP at the target locus. Importantly, mutated alleles were not only detected in T_0_, but were inherited by their descendants. Non-transgenic plants were obtained by either self-fertilization or crossing the T_0_ events with WT B104 plants. With one exception, all edited alleles observed in T_0_ events were also detected in non-transgenic T_1_ plants.

The phenotypic variation (ranging from WT green to mutant albino) we have observed in B104 and tropical line backgrounds are in accordance with the proposed roles of *VYL* and *VYL-MODIFIER*. These genes encode components of ClpP, a chloroplast-targeted protease involved in the biogenesis and function of this organelle. These genes exhibit variable degrees of functional redundancy in different maize genetic backgrounds. In the B73 inbred line, the *vyl* loss-of-function mutation results in defective thylakoid accumulation within chloroplasts and a chlorotic leaf phenotype during the seedling stage. Conversely, *vyl-modifier* loss-of-function alone does not yield any observable phenotype, but its expression is induced in the *vyl* mutant background, restoring the WT phenotype as seedlings grow. Loss of both gene functions leads to a lethal phenotype (Xing et al., 2014).

As anticipated, in our study all non-transgenic plants resulting from backcrosses exhibited the WT phenotype, as they harbor one functional *VYL* and *VYL-MODIFIER* alleles inherited from the WT B104 parent. In contrast, with two exceptions, all transgenic T_1_ plants presented the pale-yellow or albino phenotypes, indicating that the gRNA/Cas9 complex remained active and generated new somatic mutations in a transgenerational GE fashion (Impens et al., 2022). Furthermore, all sequenced albino plants also harbored indels at the *VYL-MODIFIER* gene. Finally, the somatic nature of the new mutations becomes clear in plants with a mosaic phenotype, i.e., plants with variegated leaves.

As previously reported by Aesaert et al. (2022), we also observed the off-target activity of pLAPAU17-VYL towards the *VYL* paralog *VYL-MODIFIER*. The albino phenotype was present in T_0_ plants of all genotypes, and sequencing of the tropical lines confirmed the presence of indels at the off-target site in all of these lines. Although such activity was already reported, its high frequency is unexpected. By employing a three-step strategy encompassing computational prediction, genome-wide biochemical target detection, and subsequent validation in maize plants, Young et al. (2019) have shown that in maize, off-target editing can be avoided by carefully designing sgRNAs with at least three mismatches in combination with at least one mismatch in the proximal region of the PAM, regarding possible off-target sites. In our case, the *VYL* spacer and the off-target site present three mismatches, two of them at the “seed” region. In addition, the same spacer sequence could not induce indels at the *VYL-MODIFIER* target site when used in other gene constructs lacking the MRs (Aesaert et al., 2022).

Interestingly, an increased GE efficiency was reported as a side-effect when employing *WUS2* as a MR to facilitate the genetic transformation of sorghum (*Sorghum bicolor* L.) (Che et al., 2022). Although the basis of such an increase in GE activity remains unclear, it seems to be linked to a prolonged expression of *WUS2*, as the increase in GE efficiency is less pronounced when *WUS2* is transiently expressed compared to approaches in which this gene is present in the T-DNA for stable integration. The authors, however, only evaluated the on-target activity, not exploring the impact of such an approach on potential off-targets. In this context, the tolerance to the SNP present at the *VYL* target site in CML360 and CML444 is likely due to the *WUS2* expression.

Although off-target GE in plants is of less concern than in other eukaryotic organisms (Graham et al., 2020), its occurrence is undesirable, especially when considering multigene families or conserved functional domains. Thus, further studies are required to elucidate if there is a causal effect of MR expression in the unspecific GE or if it is related to other aspects of the gene construct used.

## 5 Conclusion

We developed a platform for the transformation and GE of agronomically valuable tropical maize lines. By employing a protoplast system for construct validation and a MR-based transformation protocol, we were able to generate inheritable mutations in three tropical maize genotypes efficiently. Importantly, we noticed a higher-than-expected off-target activity, which could be related to *WUS* expression. Finally, although two of the tested lines were recalcitrant to our approach, recent advances in maize transformation, based on the usage of ternary vector systems or novel MRs such as GRF-GIF, could help to expand GE technologies to a broader range of maize germplasm.

## Data Availability

The datasets presented in this study can be found in online repositories. The names of the repository/repositories and accession number(s) can be found below: https://www.ncbi.nlm.nih.gov/bioproject/PRJNA1020177/.
