# Supplementary material for: Enabling genome editing in tropical maize lines through an improved, morphogenic regulator-assisted transformation protocol
Source: Front Genome Ed. 2023 Dec 7;5:1241035. doi: 10.3389/fgeed.2023.1241035 (PMC10748596; doi:10.3389/fgeed.2023.1241035)
Supplement: Supplementary file 1 [file Image5.PDF]

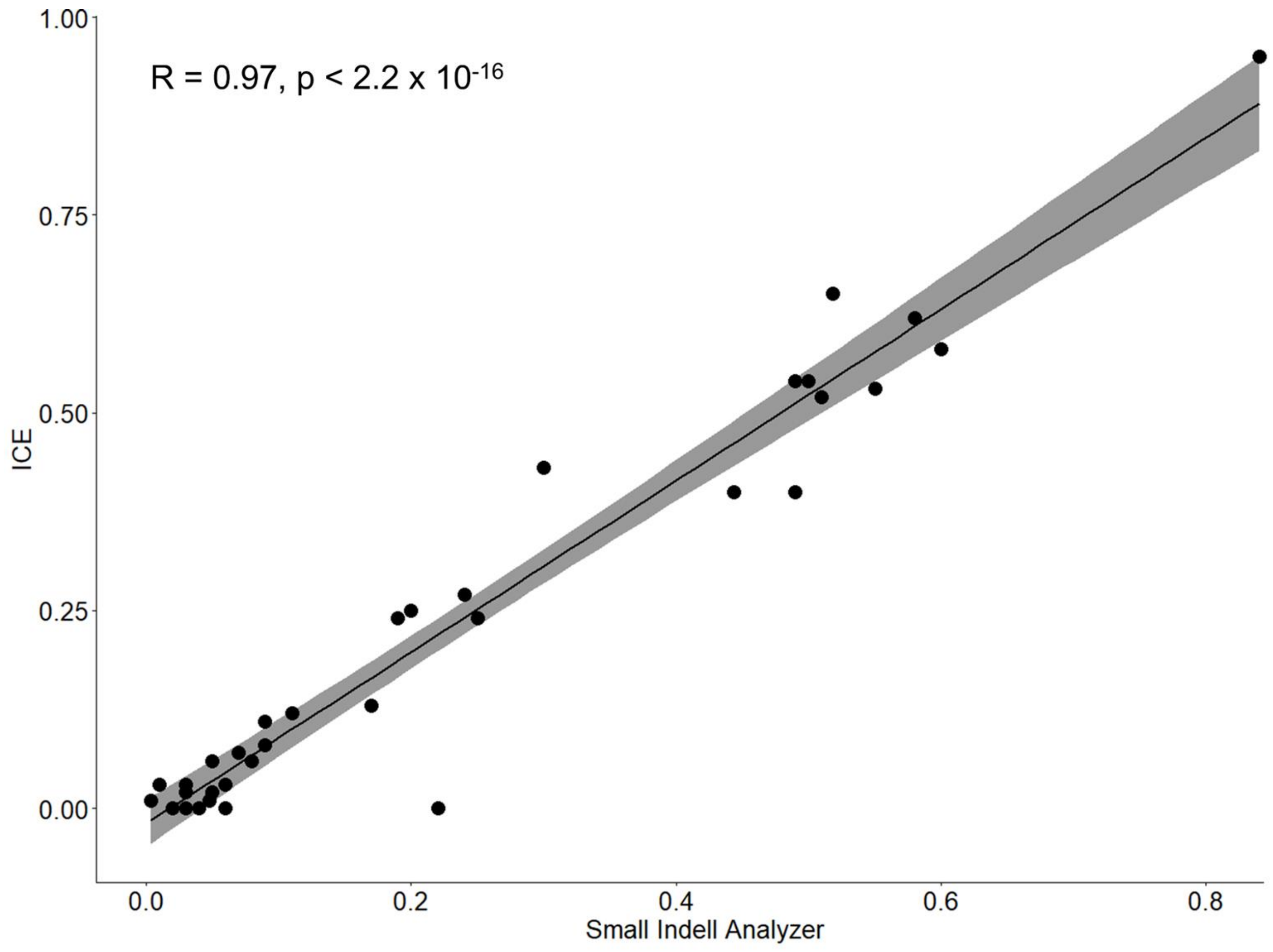

Supplementary Figure S5. Correlation of indel frequencies obtained with Small Indell Analyzer and Inference of CRISPR Edits (ICE, Synthego) tools. The Pearson correlation coefficient is used.
