# Supplementary material for: Enabling genome editing in tropical maize lines through an improved, morphogenic regulator-assisted transformation protocol
Source: Front Genome Ed. 2023 Dec 7;5:1241035. doi: 10.3389/fgeed.2023.1241035 (PMC10748596; doi:10.3389/fgeed.2023.1241035)
Supplement: Supplementary file 2 [file Table1.DOCX]

**Supplementary Table S1** – T-DNA sequence of pGC69.

LB-2xp35S-BAR-pZmUBI-GFP-RB (highlighted sequences represent introns).

GGCAGGATATATTCAATTGTAAATGGCTTCATGTCCGGGAAATCTACATGGATCAGCAATGAGTATGATGGTCAATATGGAGAAAAAGAAAGAGTAATTACCAATTTTTTTTCAATTCAAAAATGTAGATGTCCGCAGCGTTATTATAAAATGAAAGTACATTTTGATAAAACGACAAATTACGATCCGTCGTATTTATAGGCGAAAGCAATAAACAAATTATTCTAATTCGGAAATCTTTATTTCGACGTGTCTACATTCACGTCCAAATGGGGGCTTAGATGAGAAACTTCACGATCGGCTCTAGACGGGCCAACATGGTGGAGCACGACACTCTCGTCTACTCCAAGAATATCAAAGATACAGTCTCAGAAGACCAAAGGGCTATTGAGACTTTTCAACAAAGGGTAATATCGGGAAACCTCCTCGGATTCCATTGCCCAGCTATCTGTCACTTCATCAAAAGGACAGTAGAAAAGGAAGGTGGCACCTACAAATGCCATCATTGCGATAAAGGAAAGGCTATCGTTCAAGATGCCTCTGCCGACAGTGGTCCCAAAGATGGACCCCCACCCACGAGGAGCATCGTGGAAAAAGAAGACGTTCCAACCACGTCTTCAAAGCAAGTGGATTGATGTGAACATGGTGGAGCACGACACTCTCGTCTACTCCAAGAATATCAAAGATACAGTCTCAGAAGACCAAAGGGCTATTGAGACTTTTCAACAAAGGGTAATATCGGGAAACCTCCTCGGATTCCATTGCCCAGCTATCTGTCACTTCATCAAAAGGACAGTAGAAAAGGAAGGTGGCACCTACAAATGCCATCATTGCGATAAAGGAAAGGCTATCGTTCAAGATGCCTCTGCCGACAGTGGTCCCAAAGATGGACCCCCACCCACGAGGAGCATCGTGGAAAAAGAAGACCTTCCAACCACGTCTTCAAAGCAAGTGGATTGATGTGATATCTCCACTGACGTAAGGGATGACGCACAATCCCACTATCCTTCGCAAGACCCTTCCTCTATATAAGGAAGTTCATTTCATTTGGAGAGGACACGCTGAAATCACCAGTCTCTCTCTACAAATCTATCTCTCTCGAGTCCTCCACCGAAATGGGCCCCGAGCGCAGGCCCGCCGACATCCGCCGTGCCACCGAGGCCGACATGCCAGCCGTCTGCACCATCGTGAACCACTACATCGAGACCTCCACGGTCAACTTCCGCACAGAGCCGCAGGAGCCTCAGGAGTGGACCGACGACCTCGTGAGGCTGCGCGAGCGCTACCCCTGGCTCGTCGCTGAGGTGGATGGCGAGGTCGCCGGCATCGCCTACGCGGGCCCCTGGAAGGTAAGTTTCTGCTTCTACCTTTGATATATATATAATAATTATCATTAATTAGTAGTAATATAATATTTCAAATATTTTTTTCAAAATAAAAGAATGTAGTATATAGCAATTGCTTTTCTGTAGTTTATAAGTGTGTATATTTTAATTTATAACTTTTCTAATATATGACCAAAATTTGTTGATGTGCAGGCACGCAACGCCTACGACTGGACGGCGGAGAGCACCGTCTACGTGTCCCCACGCCACCAGCGCACCGGCCTGGGCTCCACGCTCTACACCCACCTGCTCAAGAGCCTGGAGGCCCAGGGCTTCAAGTCCGTGGTCGCTGTGATCGGCCTCCCCAACGACCCGAGCGTCCGTATGCACGAGGCCCTCGGCTACGCGCCCCGCGGGATGCTGAGGGCCGCGGGCTTCAAGCACGGCAACTGGCACGACGTGGGCTTCTGGCAGCTCGACTTCTCCCTGCCTGTCCCACCCCGCCCGGTGCTCCCCGTCACCGAAATCTGAACTCCGGAATCGCGTTCGAGTATTATGGCATTGGGAAAACTGTTTTTCTTGTACCATTTGTTGTGCTTGTAATTTACTGTGTTTTTTATTCGGTTTTCGCTATCGAACTGTGAAATGGAAATGGATGGAGAAGAGTTAATGAATGATATGGTCCTTTTGTTCATTCTCAAATTAATATTATTTGTTTTTTCTCTTATTTGTTGTGTGTTGAATTTGAAATTATAAGAGATATGCAAACATTTTGTTTTGAGTAAAAATGTGTCAAATCGTGGCCTCTAATGACCGAAGTTAATATGAGGAGTAAAACACTGAAGCCTGCAGGCATGCAAGCTGATCCACTAGAGGCCATGGGGGCCAATTTAAATGGCGTACGTGCAGTGCAGCGTGACCCGGTCGTGCCCCTCTCTAGAGATAATGAGCATTGCATGTCTAAGTTATAAAAAATTACCACATATTTTTTTTGTCACACTTGTTTGAAGTGCAGTTTATCTATCTTTATACATATATTTAAACTTTACTCTACGAATAATATAATCTATAGTACTACAATAATATCAGTGTTTTAGAGAATCATATAAATGAACAGTTAGACATGGTCTAAAGGACAATTGAGTATTTTGACAACAGGACTCTACAGTTTTATCTTTTTAGTGTGCATGTGTTCTCCTTTTTTTTTGCAAATAGCTTCACCTATATAATACTTCATCCATTTTATTAGTACATCCATTTAGGGTTTAGGGTTAATGGTTTTTATAGACTAATTTTTTTAGTACATCTATTTTATTCTATTTTAGCCTCTAAATTAAGAAAACTAAAACTCTATTTTAGTTTTTTTATTTAATAATTTAGATATAAAATAGAATAAAATAAAGTGACTAAAAATTAAACAAATACCCTTTAAGAAATTAAAAAAACTAAGGAAACATTTTTCTTGTTTCGAGTAGATAATGCCAGCCTGTTAAACGCCGTCGATCGACGAGTCTAACGGACACCAACCAGCGAACCAGCAGCGTCGCGTCGGGCCAAGCGAAGCAGACGGCACGGCATCTCTGTCGCTGCCTCTGGACCCCTCTCGAGAGTTCCGCTCCACCGTTGGACTTGCTCCGCTGTCGGCATCCAGAAATTGCGTGGCGGAGCGGCAGACGTGAGCCGGCACGGCAGGCGGCCTCCTCCTCCTCTCACGGCACCGGCAGCTACGGGGGATTCCTTTCCCACCGCTCCTTCGCTTTCCCTTCCTCGCCCGCCGTAATAAATAGACACCCCCTCCACACCCTCTTTCCCCAACCTCGTGTTGTTCGGAGCGCACACACACACAACCAGATCTCCCCCAAATCCACCCGTCGGCACCTCCGCTTCAAGGTACGCCGCTCGTCCTCCCCCCCCCCCCCTCTCTACCTTCTCTAGATCGGCGTTCCGGTCCATGGTTAGGGCCCGGTAGTTCTACTTCTGTTCATGTTTGTGTTAGATCCGTGTTTGTGTTAGATCCGTGCTGCTAGCGTTCGTACACGGATGCGACCTGTACGTCAGACACGTTCTGATTGCTAACTTGCCAGTGTTTCTCTTTGGGGAATCCTGGGATGGCTCTAGCCGTTCCGCAGACGGGATCGATCTAGGATAGGTATACATGTTGATGTGGGTTTTACTGATGCATATACATGATGGCATATGCAGCATCTATTCATATGCTCTAACCTTGAGTACCTATCTATTATAATAAACAAGTATGTTTTATAATTATTTTGATCTTGATATACTTGGATGATGGCATATGCAGCAGCTATATGTGGATTTTTTTAGCCCTGCCTTCATACGCTATTTATTTGCTTGGTACTGTTTCTTTTGTCGATGCTCACCCTGTTGTTTGGTGTTACTTCTGCAGGTACTAGTGGATCCCCCGGGCTGCAGGAATTCAAGCTTACGCGTCCTCCACCGAAATGGGCAAGGGCGAGGAACTGTTCACTGGCGTGGTCCCAATCCTGGTGGAACTGGATGGTGATGTGAACGGGCACAAGTTCTCCGTCAGCGGAGAGGGTGAAGGTGATGCCACCTACGGAAAGCTCACCCTGAAGTTCATCTGCACTACCGGAAAGCTCCCTGTTCCGTGGCCAACCCTCGTCACCACTTTCACCTACGGTGTTCAGTGCTTCTCCCGGTACCCAGATCACATGAAGCAGCATGACTTCTTCAAGAGCGCCATGCCCGAAGGCTACGTGCAAGAAAGGACTATCTTCTTCAAGGATGACGGGAACTACAAGACACGTGCCGAAGTCAAGTTCGAAGGTGATACCCTGGTGAACCGCATCGAGCTGAAAGGTAAGTTTCTGCTTCTACCTTTGATATATATATAATAATTATCATTAATTAGTAGTAATATAATATTTCAAATATTTTTTTCAAAATAAAAGAATGTAGTATATAGCAATTGCTTTTCTGTAGTTTATAAGTGTGTATATTTTAATTTATAACTTTTCTAATATATGACCAAAATTTGTTGATGTGCAGGTATCGATTTCAAGGAAGATGGAAACATCCTCGGACACAAGCTGGAGTACAACTACAACTCCCACAACGTATACATCATGGCCGACAAGCAGAAGAACGGCATCAAGGTGAACTTCAAGATCAGGCACAACATCGAAGATGGAAGCGTGCAACTGGCGGACCACTACCAGCAGAACACGCCCATCGGCGATGGCCCTGTCCTGCTGCCGGACAACCATTACCTGTCCACGCAATCTGCCCTCTCCAAGGACCCCAACGAGAAGAGGGACCACATGGTCCTGCTGGAGTTCGTGACGGCTGCTGGGATCACGCATGGCATGGATGAACTCTACAAGTGACTCGTCGACTCGAATTTCCCCGATCGTTCAAACATTTGGCAATAAAGTTTCTTAAGATTGAATCCTGTTGCCGGTCTTGCGATGATTATCATATAATTTCTGTTGAATTACGTTAAGCATGTAATAATTAACATGTAATGCATGACGTTATTTATGAGATGGGTTTTTATGATTAGAGTCCCGCAATTATACATTTAATACGCGATAGAAAACAAAATATAGCGCGCAAACTAGGATAAATTATCGCGCGCGGTGTCATCTATGTTACTAGATCGCTCGACGCATGCTGGTACCTGACGTCTGCGGCCGCATTAATTAAGGCCCTAAGGGCCAGATCTTGGGCCCGGTACCCGATCAGATTGTCGTTTCCCGCCTTCGGTTTAAACTATCAGTGTTTGACAGGATATATTGGCGGGTAAAC

**Supplementary Table S2** – Primers used in this study.

| Name | Description | Sequence 5’ – 3’ | Usage |
| --- | --- | --- | --- |
| Pr140 | sgRNA module_Fw | TGCCTTTTCTTATCGACCATGT | Presence of T-DNA |
| Pr141 | sgRNA module_Rv | GTGGTTGATGGGTTGATTGCT |  |
| Pr182 | VYL_Rv | CCATTCCTAACAGGAAACGAA | VYL genotyping |
| Pr183 | VYL_Fw | GGGCTTGTTTGGTTGGATACT |  |
| Pr211 | ZmVYL_NGS_Fw1 | TCTCCCCTCAACCGAACAAAG | VYL deep amplicon sequencing |
| Pr212 | ZmVYL_NGS_Rv2 | TACAGCAGCAAACGGTTGTC |  |
| Pr184 | VYL-MODIFIER_Rv | TAGAGTGAATATGACTGCAGAGG | VYL-MODIFIER genotyping |
| Pr185 | VYL-MODIFIER_Fw | GATTTGGTCGATCAGGTGGAAT |  |
| Pr213 | ZmVYL-paral_NGS_Fw2 | CAGCACGTACGATGGCACTA | VYL-MODIFIER deep amplicon sequencing |
| Pr214 | ZmVYL-paral_NGS_Rv2 | CCTTCGTTCTCACCTTAGTGGG |  |
| Pr186 | pBdEF1a_Fw | AAAGAATGGTGGGGTCCACG | MRs excision |
| Pr188 | mRuby_Rv | TGAGTTCCCTCGTACGGTCT |  |
| Pr209 | pAXIG1_Fw | CTCACGGGTAGCTCATGGTC | MRs presence |
| Pr210 | WUS2_Rv | GACGACAGCGAGAGGACG |  |

**Supplementary table S3** – Detailed information on each transformation experiment. T_0_ total indicates transgenic regenerants. Efficiency is calculated as the T_0_ total divided by the starting number of IZEs. DAP – days after pollination.

|  | |  |  |  |  |  |  |  |  |
| --- | --- | --- | --- | --- | --- | --- | --- | --- | --- |
| **Genotype** | **Date** | | **Ear** | **DAP** | **Starting IZEs** | **Regenerated Plants** | **T_0_ total** | **Transformation Efficiency** | **Efficiency / line** |
| B104 | 05/05/2021 | | I | 16d | 201 | 0 | 0 | 0,00% |  |
|  |  |  | II | 16d | 239 | 0 | 0 | 0,00% |  |
|  |  |  | III | 16d | 234 | 0 | 0 | 0,00% |  |
|  |  |  | IV | 16d | 131 | 13 | 13 | 9,92% |  |
|  |  |  | V | 16d | 256 | 2 | 2 | 0,78% |  |
|  |  |  | VI | 16d | 146 | 16 | 16 | 10,96% |  |
|  |  |  | VII | 16d | 139 | 0 | 0 | 0,00% |  |
|  |  |  | VIII | 16d | 191 | 0 | 0 | 0,00% |  |
|  | **Total** | | **8** |  | **1537** | **31** | **31** | **2,02%** | **2,02%** |
| CML360 | 05/04/2022 | | I | 12d | 215 | 0 | 0 | 0,00% |  |
|  |  |  | II | 12d | 145 | 0 | 0 | 0,00% |  |
|  |  |  | III | 12d | 217 | 6 | 5 | 2,30% |  |
|  |  |  | IV | 12d | 154 | 0 | 0 | 0,00% |  |
|  |  |  | V | 12d | 182 | 0 | 0 | 0,00% |  |
|  | **Total** | | **5** |  | **913** | **6** | **5** | **0,55%** |  |
|  | 11/11/2022 | | I | 15d | 141 | 4 | 3 | 2,13% |  |
|  |  |  | II | 15d | 123 | 2 | 1 | 0,81% |  |
|  |  |  | III | 15d | 129 | 3 | 2 | 1,55% |  |
|  |  |  | IV | 15d | 147 | 0 | 0 | 0,00% |  |
|  |  |  | V | 15d | 135 | 0 | 0 | 0,00% |  |
|  |  |  | VI | 15d | 101 | 0 | 0 | 0,00% |  |
|  | **Total** | | **6** |  | **776** | **9** | **6** | **0,77%** | **0,65%** |
| CML444 | 15/12/2021 | | I | 14d | 191 | 10 | 10 | 5,24% |  |
|  | **Total** | | **1** |  | **191** | **10** | **10** | **5,24%** |  |
|  | 18/12/2021 | | III | 12d | 211 | 5 | 4 | 1,90% |  |
|  |  |  | IV | 12d | 130 | 4 | 4 | 3,08% |  |
|  | **Total** | | **2** |  | **341** | **9** | **8** | **2,35%** | **3,38%** |
| CML488 | 18/12/2021 | | I | 13d | 105 | 0 | 0 | 0,00% |  |
|  |  |  | II | 13d | 62 | 0 | 0 | 0,00% |  |
|  | **Total** | | **2** |  | **167** | **0** | **0** | **0,00%** |  |
|  | 12/04/2022 | | I | 12d | 118 | 0 | 0 | 0,00% |  |
|  |  |  | II | 12d | 148 | 0 | 0 | 0,00% |  |
|  |  |  | III | 12d | 148 | 0 | 0 | 0,00% |  |
|  |  |  | IV | 12d | 116 | 0 | 0 | 0,00% |  |
|  |  |  | V | 12d | 90 | 0 | 0 | 0,00% |  |
|  |  |  | VI | 12d | 70 | 0 | 0 | 0,00% |  |
|  |  |  | VII | 12d | 27 | 0 | 0 | 0,00% |  |
|  | **Total** | | **7** |  | **717** | **0** | **0** | **0,00%** | **0,00%** |
| PCL1 | 25/02/2022 | | I | NA | 25 | 0 | 0 | 0,00% |  |
|  |  |  | II | NA | 85 | 12 | 12 | 14,12% |  |
|  |  |  | III | NA | 71 | 0 | 0 | 0,00% |  |
|  | **Total** | | **3** |  | **181** | **12** | **12** | **6,63%** | **6,63%** |
| PCL2 | 25/02/2022 | | I | NA | 34 | 0 | 0 | 0,00% |  |
|  |  |  | II | NA | 98 | 0 | 0 | 0,00% |  |
|  |  |  | III | NA | 47 | 0 | 0 | 0,00% |  |
|  |  |  | IV | NA | 39 | 0 | 0 | 0,00% |  |
|  | **Total** | | **4** |  | **218** | **0** | **0** | **0,00%** | **0,00%** |

**Supplementary table S4** – Detailed information on UBQ:RUBY transient expression in immature zygotic embryos (IZEs) as observed three days after *Agrobacterium* infection. DAP – days after pollination.

| **Genotype** | **Date** | **Ear** | **DAP** | **IZEs** | **RUBY +** | **%** | **Average / line** |
| --- | --- | --- | --- | --- | --- | --- | --- |
| CML360 | 11/11/2022 | I | 15d | 26 | 21 | 80,77% |  |
|  |  | II | 15d | 29 | 19 | 65,52% |  |
|  |  | III | 15d | 23 | 15 | 65,22% |  |
|  |  | IV | 15d | 28 | 15 | 53,57% |  |
|  |  | V | 15d | 23 | 7 | 30,43% |  |
|  |  | VI | 15d | 17 | 9 | 52,94% |  |
|  | **Total** | **6** |  | **146** | **86** | **58,90%** | **58,90%** |
| CML488 | 12/04/2022 | I | 12d | 25 | 0 | 0,00% |  |
|  |  | II | 12d | 22 | 0 | 0,00% |  |
|  |  | III | 12d | 21 | 0 | 0,00% |  |
|  |  | IV | 12d | 17 | 0 | 0,00% |  |
|  |  | V | 12d | 17 | 0 | 0,00% |  |
|  |  | VI | 12d | 14 | 0 | 0,00% |  |
|  |  | VII | - | - | - | - |  |
|  | **Total** | **7** |  | **116** | **0** | **0,00%** | **0,00%** |
| PCL1 | 25/02/2022 | I | 13 | 5 | 0 | 0,00% |  |
|  |  | II | 13 | 15 | 5 | 33,33% |  |
|  |  | III | 13 | 9 | 0 | 0,00% |  |
|  | **Total** | **3** |  | **29** | **5** | **17,24%** | **17,24%** |
| PCL2 | 25/02/2022 | I | 13 | 10 | 0 | 0,00% |  |
|  |  | II | 13 | 14 | 0 | 0,00% |  |
|  |  | III | 13 | 22 | 0 | 0,00% |  |
|  |  | IV | 13 | 13 | 0 | 0,00% |  |
|  | **Total** | **4** |  | **59** | **0** | **0,00%** | **0,00%** |

**Supplementary table S5** – Genotyping of T_0_ plants of B104 and tropical maize lines, including the presence of sgRNA, the morphogenic regulators (MR) cassette and the major indels found at the *VYL* target site, with respective frequency found in ICE analysis. Different colors in the second to fourth columns indicate independent genome editing events. Green color in the MR cassette columns indicates full excision of the MRs.

|  |  |  |  |  | **MR cassette** | |
| --- | --- | --- | --- | --- | --- | --- |
| **Line** | **Event ID** | **Indels** | **Zygosity** | **sgRNA** | **Excision** | **WUS2** |
| B104 | 61GC102_IV_1a | -20 (55%) / +1 (42%) | biallelic | + | + | + |
|  | 61GC102_IV_1b | -20 (60%) / +1 (36%) | biallelic | + | - | + |
|  | 61GC102_IV_1c | -20 (52%) / +1 (42%) | biallelic | + | + | + |
|  | 61GC102_IV_2a | +1 (99%) | homozygous | + | + | + |
|  | 61GC102_IV_3a | -29 (49%) / -4 (48%) | biallelic | + | + | - |
|  | 61GC102_IV_4a | +1 (89%) | homozygous | + | - | + |
|  | 61GC102_IV_4b | -25 (28%) / -2 (70%) | homozygous | + | - | + |
|  | 61GC102_IV_5a | -1 (50%) / 0 (26%) / +1 (22%) | mosaic | + | + | + |
|  | 61GC102_IV_5b | -1 (18%) / +1 (79%) | homozygous | + | - | + |
|  | 61GC102_IV_6a | +1 (92%) | homozygous | + | + | + |
|  | 61GC102_IV_7a | -2 (25%) / +1(56%) | mosaic | + | + | + |
|  | 61GC102_IV_8a | +1 (97%) | homozygous | + | + | - |
|  | 61GC102_IV_9a | +1 (99%) | homozygous | + | + | + |
|  | 62GC102_V_2a | +1 (98%) | homozygous | + | + | + |
|  | 62GC102_VI_1a | +1 (96%) | homozygous | + | + | + |
|  | 62GC102_VI_1b | -25 (98%) | homozygous | + | + | - |
|  | 62GC102_VI_1c | -14 (99%) | homozygous | + | + | - |
|  | 62GC102_VI_2a | +1 (65%) / multiple indels | mosaic | + | + | + |
|  | 62GC102_VI_2b | +1 (37%) / multiple indels | mosaic | + | - | + |
|  | 62GC102_VI_2c | +1 (89%) | homozygous | + | - | + |
|  | 62GC102_VI_3a | -11 (56%) / +1 (41%) | biallelic | + | + | + |
|  | 62GC102_VI_4a | 0 (52%) / +1 (45%) | heterozygous | + | + | + |
|  | 62GC102_VI_4b | +1 (35%) / multiple indels | mosaic | + | + | + |
|  | 62GC102_VI_6a | +1 (98%) | homozygous | + | - | + |
|  | 62GC102_VI_6b | -3 (14%) / +1 (77%) | homozygous | + | - | + |
|  | 62GC102_VI_7a | -26 (36%) / -4 (29%) | mosaic | + | + | + |
|  | 62GC102_VI_8a | -15 (53%) / +1 (44%) | biallelic | + | + | + |
|  | 62GC102_VI_9a | -3 (99%) | homozygous | + | + | + |
|  | 62GC102_VI_9b | +1 (99%) | homozygous | + | + | + |
| CML360 | 3CML_III_1a | -6 (43%) / 0 (46%) | heterozygous | + | + | + |
|  | 3CML_III_1b | 0 (39%) / +1 (53%) | heterozygous | + | + | - |
|  | 3CML_III_1c | 0 (32%) / +1 (52%) | heterozygous | + | + | + |
|  | 3CML_III_1d | -1 (11%) / 0 (22%) / +1 (62%) | mosaic | + | + | + |
|  | 3CML_III_3a | -2 (54%) / 0 (34%) | heterozygous | + | + | + |
|  | 5CML_I_1a | 0 (34%) / +1 (56%) | heterozygous | + | + | - |
|  | 5CML_I_1b | -1 (50%) / +1 (47%) | biallelic | + | + | + |
|  | 5CML_I_2a | NA |  | + | + | + |
|  | 5CML_II_2a | +1 (96%) | homozygous | + | + | - |
|  | 5CML_III_1a | NA |  | + | - | + |
|  | 5CML_III_1d | 0 (88%) | WT | + | - | + |
| CML444 | 1CML_I_1a | 0 (50%) / +1 (40%) | heterozygous | + | + | - |
|  | 1CML_I_1b | -1 (40%) / 0 (52%) | heterozygous | + | + | + |
|  | 1CML_I_2a | +1 (95%) | homozygous | + | + | - |
|  | 1CML_I_3a | -5 (25%) / -1 (12%) / 0 (58%) | mosaic | + | + | - |
|  | 1CML_I_4a | 0 (74%) / +1 (24%) | mosaic | + | + | - |
|  | 1CML_I_5a | 0 (86%) / +1 (13%) | WT | + | + | - |
|  | 1CML_I_6a | 0 (31%) / +1 (58%) | heterozygous | + | + | - |
|  | 1CML_I_7a | 0 (38%) / +1 (57%) | heterozygous | + | + | + |
|  | 1CML_I_8a | 0 (37%) / +1 (57%) | heterozygous | + | + | + |
|  | 1CML_I_9a | 0 (42%) / +1 (50%) | heterozygous | + | - | - |
|  | 2CML_III_2a | -1 (57%) / 0 (20%) / +1 (19%) | mosaic | + | + | - |
|  | 2CML_III_3a | -55 (NA) | homozygous | + | + | + |
|  | 2CML_III_4a | -1 (24%) / 0 (46%) / +1 (27%) | mosaic | + | + | + |
|  | 2CML_III_5a | 0 (42%) / +1 (53%) | heterozygous | + | + | + |
|  | 2CML_IV_1a | Large indel | NA | + | + | - |
|  | 2CML_IV_1b | Large indel | NA | + | + | + |
|  | 2CML_IV_1c | Large indel | NA | + | + | + |
|  | 2CML_IV_2a | -3 (64%) / 0 (34%) | heterozygous | + | + | + |
| PCL1 | 1PCL_II_1a.1 | -3 (100%) | homozygous | + | + | - |
|  | 1PCL_II_1a.2 | -1 (48%) / +1 (50%) | biallelic | + | + | - |
|  | 1PCL_II_1a.3 | -1 (47%) / +1 (49%) | biallelic | + | + | + |
|  | 1PCL_II_1a.4 | -1 (47%) / +1 (49%) | biallelic | + | + | - |
|  | 1PCL_II_1a.5 | -3 (61%) / +1 (29%) | mosaic | + | + | - |
|  | 1PCL_II_2a | +1 (98%) | homozygous | + | + | - |
|  | 1PCL_II_4a | -1 (50%) / +1 (46%) | biallelic | + | + | + |
|  | 1PCL_II_5a | -14 (52%) / +1 (45%) | biallelic | + | + | - |
|  | 1PCL_II_6a | -8 (52%) / -1 (43%) | biallelic | + | + | - |
|  | 1PCL_II_7a.1 | -3 (31%) / -2 (29%) / +1 (24%) | mosaic | + | + | + |
|  | 1PCL_II_7a.2 | +1 (78%) / +2 (18%) | homozygous | + | + | + |
|  | 1PCL_II_8a | +1 (97%) | homozygous | + | + | - |
