# Supplementary material for: Enabling genome editing in tropical maize lines through an improved, morphogenic regulator-assisted transformation protocol
Source: Front Genome Ed. 2023 Dec 7;5:1241035. doi: 10.3389/fgeed.2023.1241035 (PMC10748596; doi:10.3389/fgeed.2023.1241035)
Supplement: Supplementary file 3 [file Image4.PDF]

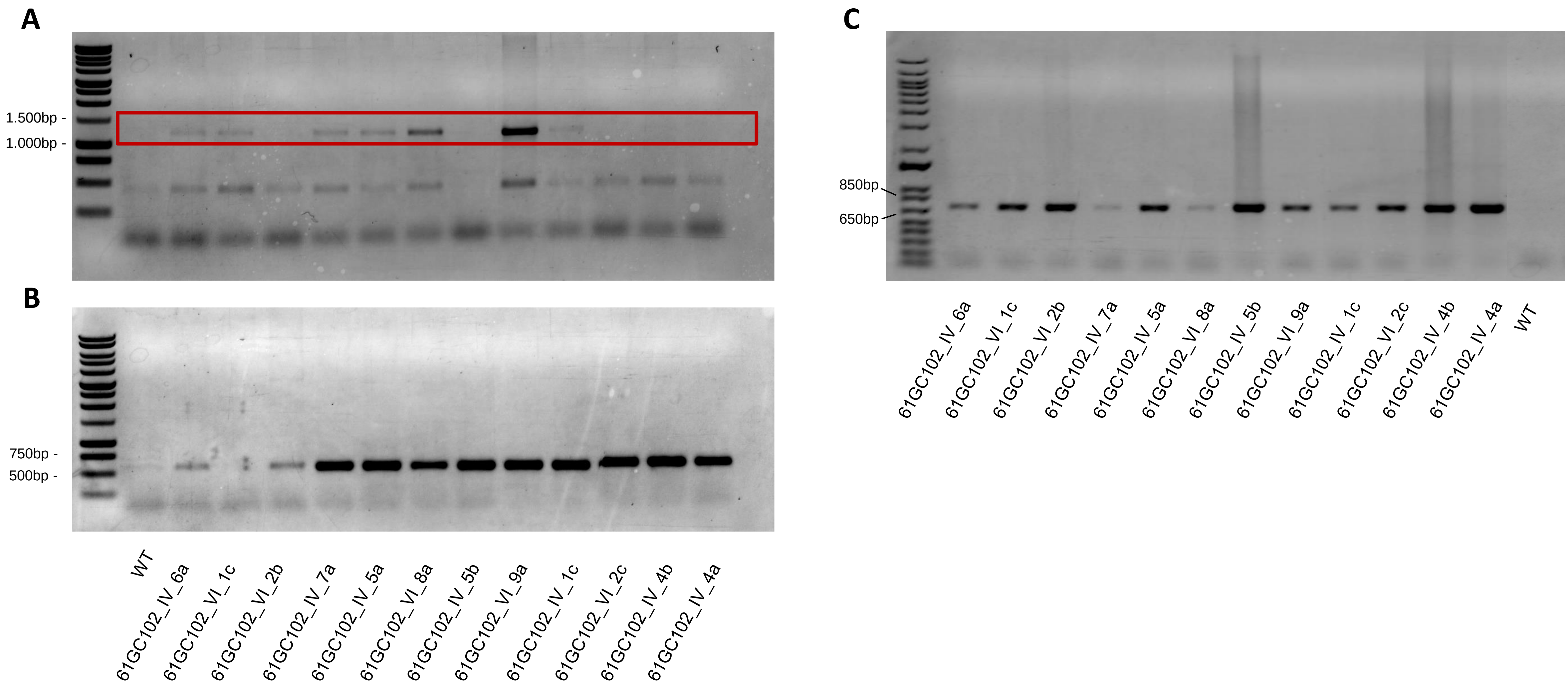

Supplementary Figure S4. Representative PCR-based genotyping of T_0 events. (A) Primers Pr186 and Pr188 produce a 1,202-bp amplicon (red box) upon excision of the MR cassette. (B) Primers Pr209 and Pr210 produce a 565-bp amplicon indicating the presence of the WUS2 MR. (C) Primers Pr140 and Pr141 produce a 739-bp amplicon indicating the presence of the sgRNA region of the T-DNA.
