# Supplementary material for: Enabling genome editing in tropical maize lines through an improved, morphogenic regulator-assisted transformation protocol
Source: Front Genome Ed. 2023 Dec 7;5:1241035. doi: 10.3389/fgeed.2023.1241035 (PMC10748596; doi:10.3389/fgeed.2023.1241035)
Supplement: Supplementary file 4 [file Image2.PDF]

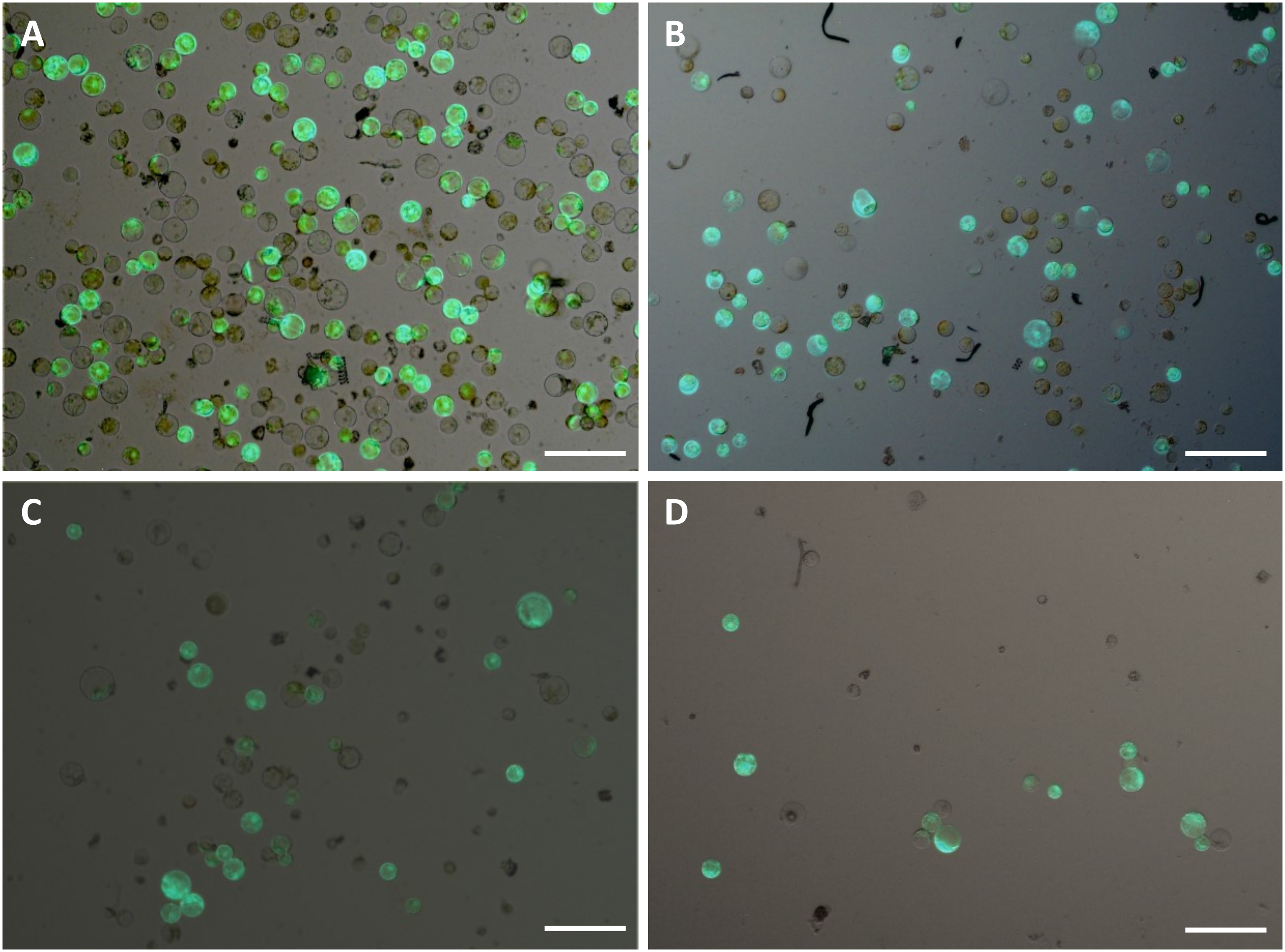

Supplementary Figure S2. Overlay of bright field and green channel images of protoplasts prepared from different genotypes and transfected with pGC69 (GFP expression cassette). **(A)** B104. **(B)**CML360. **(C)**CML444. **(D)**PCL1.
