# Supplementary material for: Enabling genome editing in tropical maize lines through an improved, morphogenic regulator-assisted transformation protocol
Source: Front Genome Ed. 2023 Dec 7;5:1241035. doi: 10.3389/fgeed.2023.1241035 (PMC10748596; doi:10.3389/fgeed.2023.1241035)
Supplement: Supplementary file 5 [file Image3.PDF]

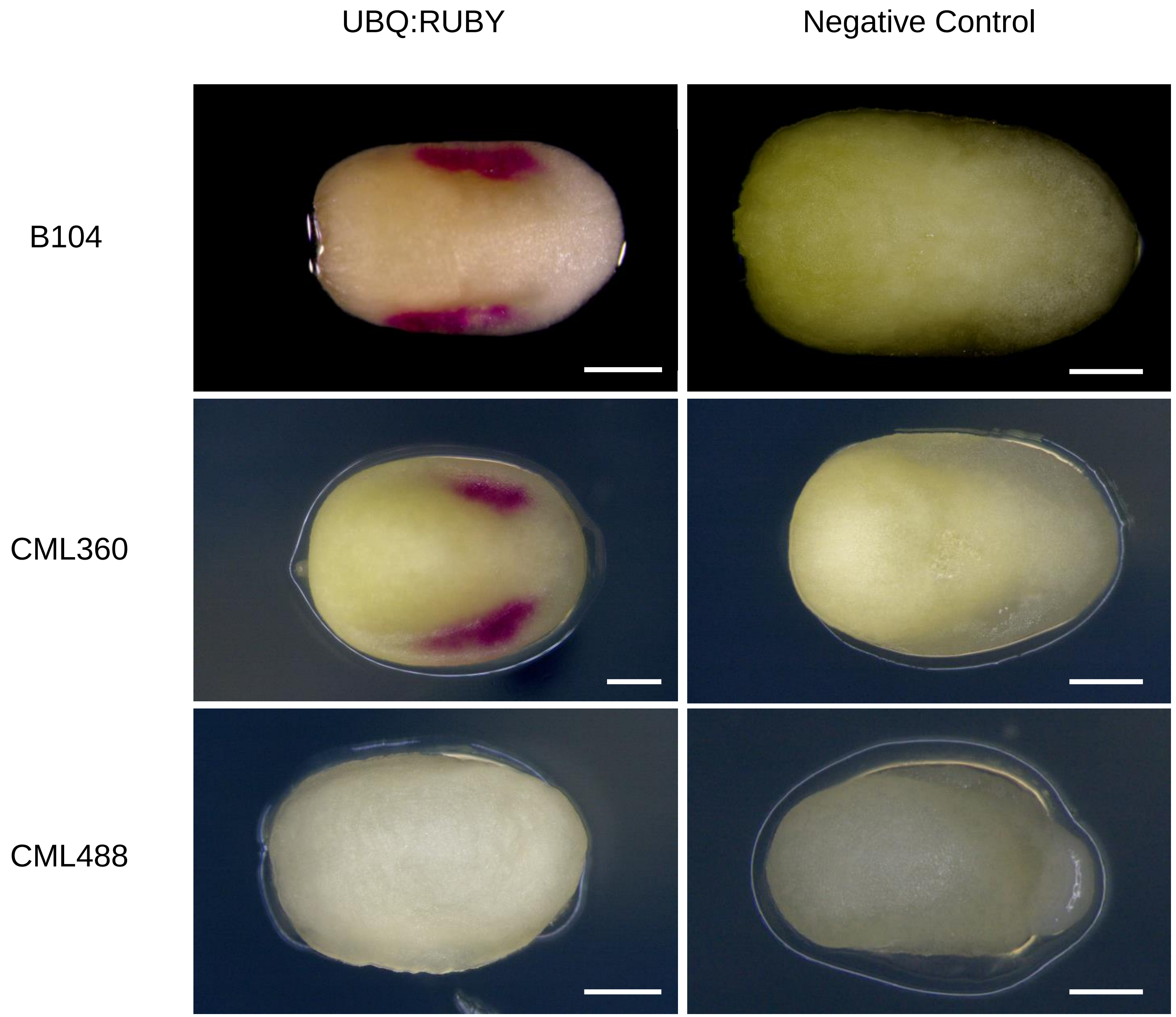

Supplementary Figure S3. Maize immature zygotic embryos transformed with UBQ:RUBY construct (left column) and their corresponding negative controls (right column), three days after *Agrobacterium* infection. Scale bar = 1mm
