# Supplementary material for: Enabling genome editing in tropical maize lines through an improved, morphogenic regulator-assisted transformation protocol
Source: Front Genome Ed. 2023 Dec 7;5:1241035. doi: 10.3389/fgeed.2023.1241035 (PMC10748596; doi:10.3389/fgeed.2023.1241035)
Supplement: Supplementary file 6 [file Image1.PDF]

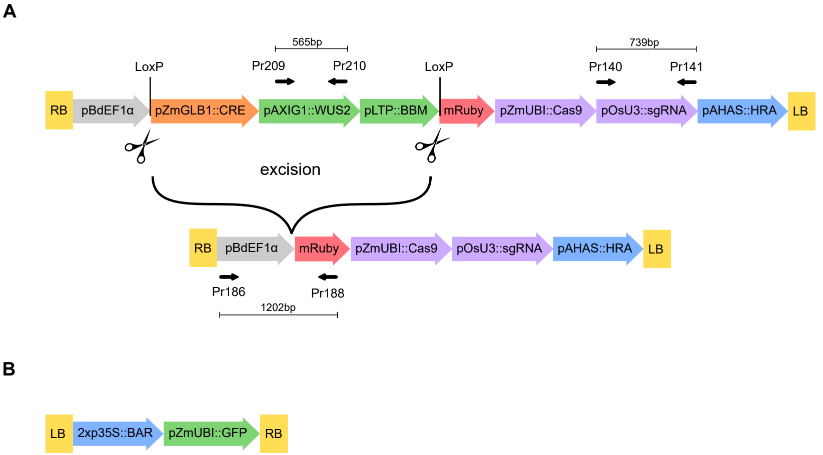

Supplementary Figure S1. Vector design. **(A)** pLAPAU17-VYL T-DNA showing expression cassettes, primer positions and the expected arrangement of T-DNA after excision of the morphogenic regulators cassette by the Cre/LoxP system. **(B)** pGC69 T-DNA showing BAR and GFP expression cassettes.
